# Supplementary material for: Lysosomal protein transmembrane 5 promotes lung-specific metastasis by regulating BMPR1A lysosomal degradation
Source: Nat Commun. 2022 Jul 16;13:4141. doi: 10.1038/s41467-022-31783-6 (PMC9288479; doi:10.1038/s41467-022-31783-6)
Supplement: Supplementary file 1 — Supplementary Information [file 41467_2022_31783_MOESM1_ESM.pdf]

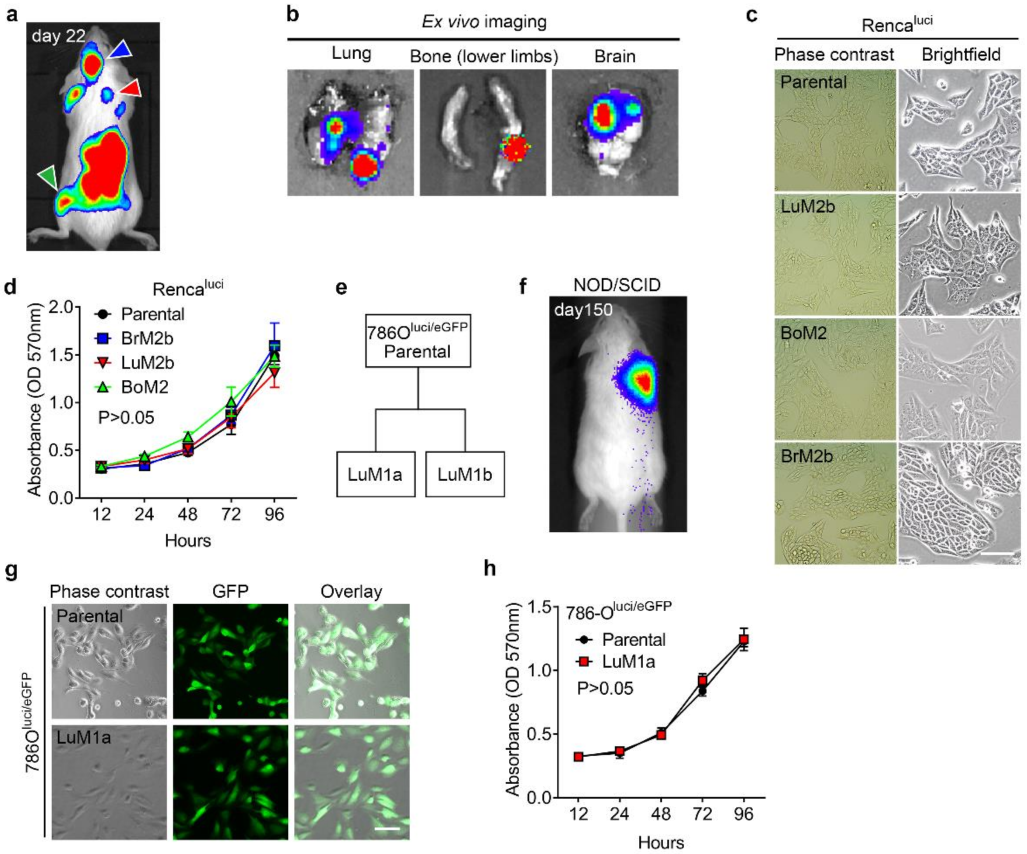

Supplementary Fig. 1 Isolation and characterization of lung metastatic derivatives

(a) Representative bioluminescence image of BALB/c mice with lung (red arrowhead), bone (green arrowhead) and brain (blue arrowhead) metastases by Renca^{luci} cells at 22 days after intracardiac inoculation.

(b) Representative *ex vivo* bioluminescent images of lung, bone (lower limbs) and brain metastases by Renca^{luci} cells after intracardiac inoculation.

(c) Representative morphology images of Renca^{luci} parental and derivative cells. Scale bar, 50 μm.

(d) MTT assay of Renca^{luci} parental and derivative cells (n=6 per group).

(e) Flowchart of the *in vivo* selection of lung metastatic subpopulations in 786O$^{luci/eGFP}$ cells.

(f) Representative bioluminescence image of NOD/SCID mice with lung metastasis by 786O$^{luci/eGFP}$ cells at 150 days after intracardiac inoculation.

(g) Representative morphology and fluorescent images of 786O$^{luci/eGFP}$ parental and LuM1a cells. Scale bar, 50 μm.

(h) MTT assay of 786O$^{luci/eGFP}$ parental and LuM1a cells (n=6 per group).

In (d) and (h), the data represent the mean ± SD. Two-way ANOVA test was used for statistical analysis in (d) and (h). Source data are provided as a Source data file.

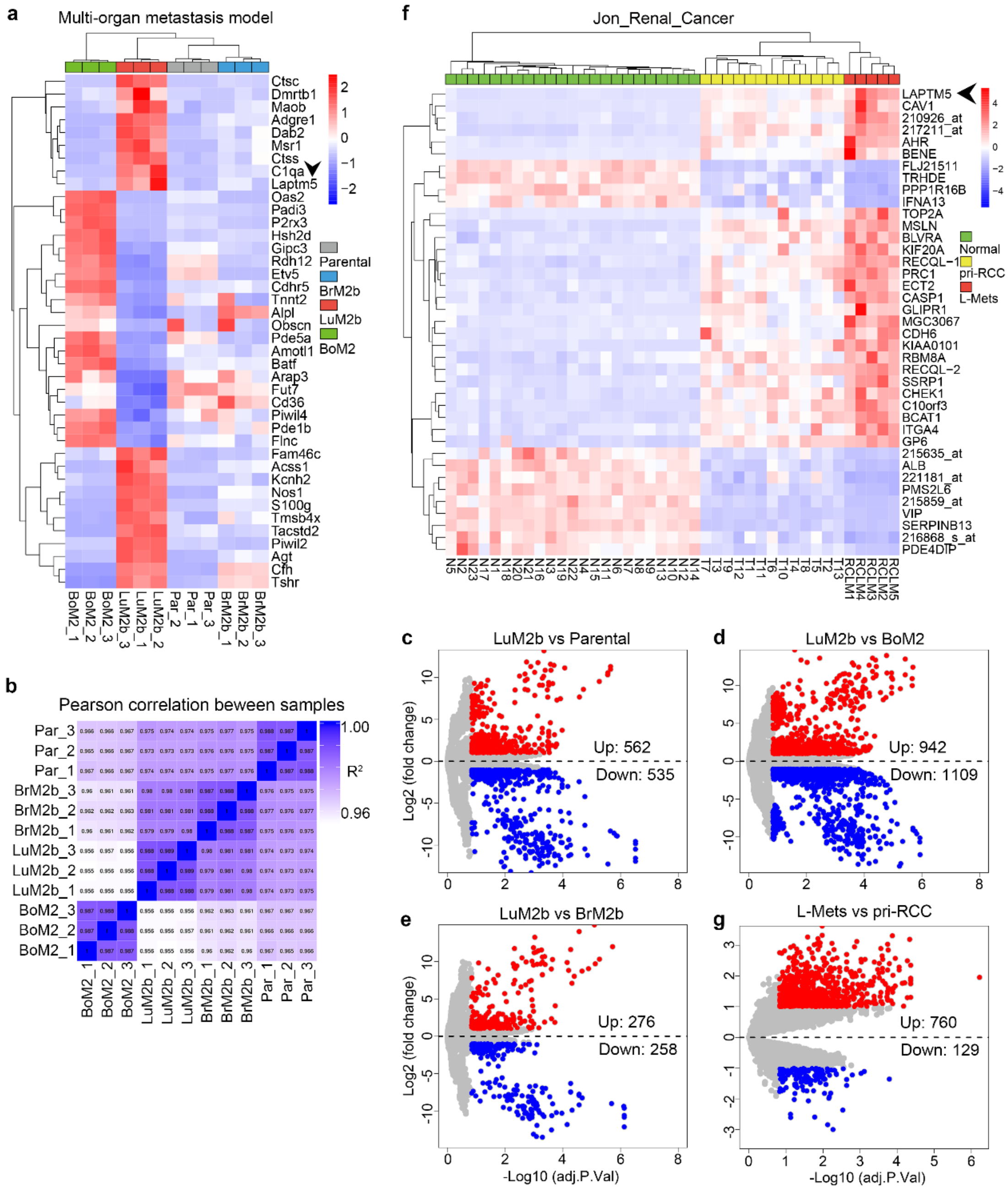

Supplementary Fig. 2 Identification of molecules associated with lung metastatic phenotypes

(a) Double clustering heatmap of top 20 up- and down-regulated genes in Renca^{LuM2b} cells as compared with Renca^{Parental}, Renca^{BrM2b} and Renca^{BoM2} cells.

(b) Visual graph of Pearson correlation between Renca^{luci} parental and derivative cells.

(c-e) Volcano plots of transcriptome sequencing results. (c), LuM2b versus parental; (d), LuM2b versus BoM2; (e), LuM2b versus BrM2b. Red dots and blue dots represent upregulated [Log_2 (fold change) > 1, -Log_{10} (adj.P.Val) > 1] and downregulated [Log_2 (fold change) < -1, -Log_{10} (adj.P.Val) > 1] genes, respectively.

(f) Double clustering heatmap of transcriptome analyses of normal kidney tissues (Normal), primary RCC without lung metastases (pri-RCC) and RCC lung metastases

(L-Mets) in the Jon_Renal_Cancer dataset.
(g) Volcano plot of L-Mets compared with pri-RCC in the Jon_Renal_Cancer dataset.
Adjustments were made for multiple comparisons in all panels.

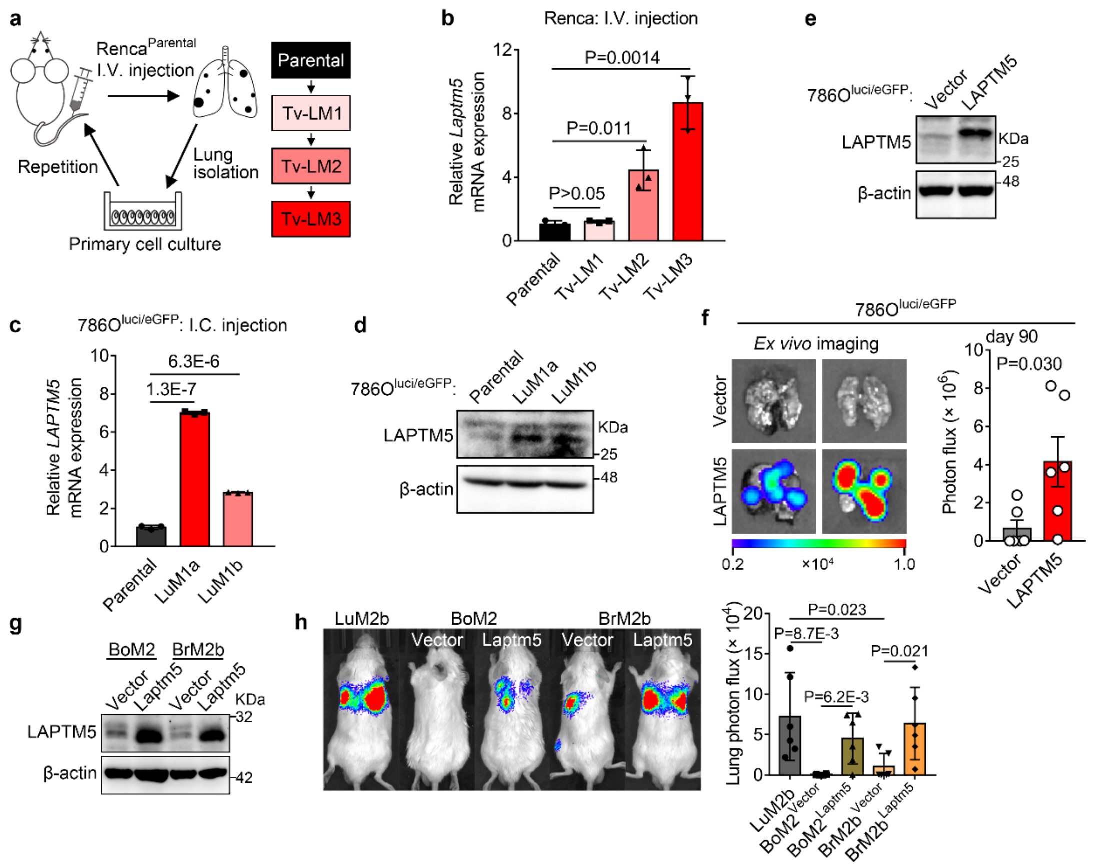

Supplementary Fig. 3 LAPTM5 mediates lung-specific metastasis

(a) Screening of highly lung-metastatic subpopulations of Renca cells through tail vein injection. I.V., intravenous.

(b) qRT-PCR analysis of *Laptm5* expression in Renca parental and derivative cells derived from (A). n=3 per group.

(c and d) qRT-PCR (c, n=3 per group) and IB (d) analysis of LAPTM5 expression in $786O^{luci/eGFP}$ parental and lung derivative cells.

(e) IB analyses of LAPTM5 in control and LAPTM5-overexpressing $786O^{luci/eGFP}$ cells.

(f) Representative *ex vivo* bioluminescent images of lung metastases at 90 days after intracardiac injection with control or LAPTM5-overexpressing $786O^{luci/eGFP}$ cells (left panel, n=6 mice per group) and quantification of the photon flux (right panel). The data represent the mean ± SEM.

(g) IB analyses of LAPTM5 in control and Laptm5-overexpressing $Renca^{BoM2}$ and $Renca^{BrM2b}$ cells.

(h) Representative *ex vivo* bioluminescent images of lung metastases at 21 days through I.V. injection with control and Laptm5-overexpressing Renca BoM2 and BrM2b cells (left panel) and quantification of the photon flux (right panel, n=6 mice per group).

Immunoblots are representative of three biological replicates. In (b), (c), and (h), the data represent the mean ± SD. Two-tailed Student's unpaired *t*-test was used for statistical analysis in all panels. Source data are provided as a Source data file.

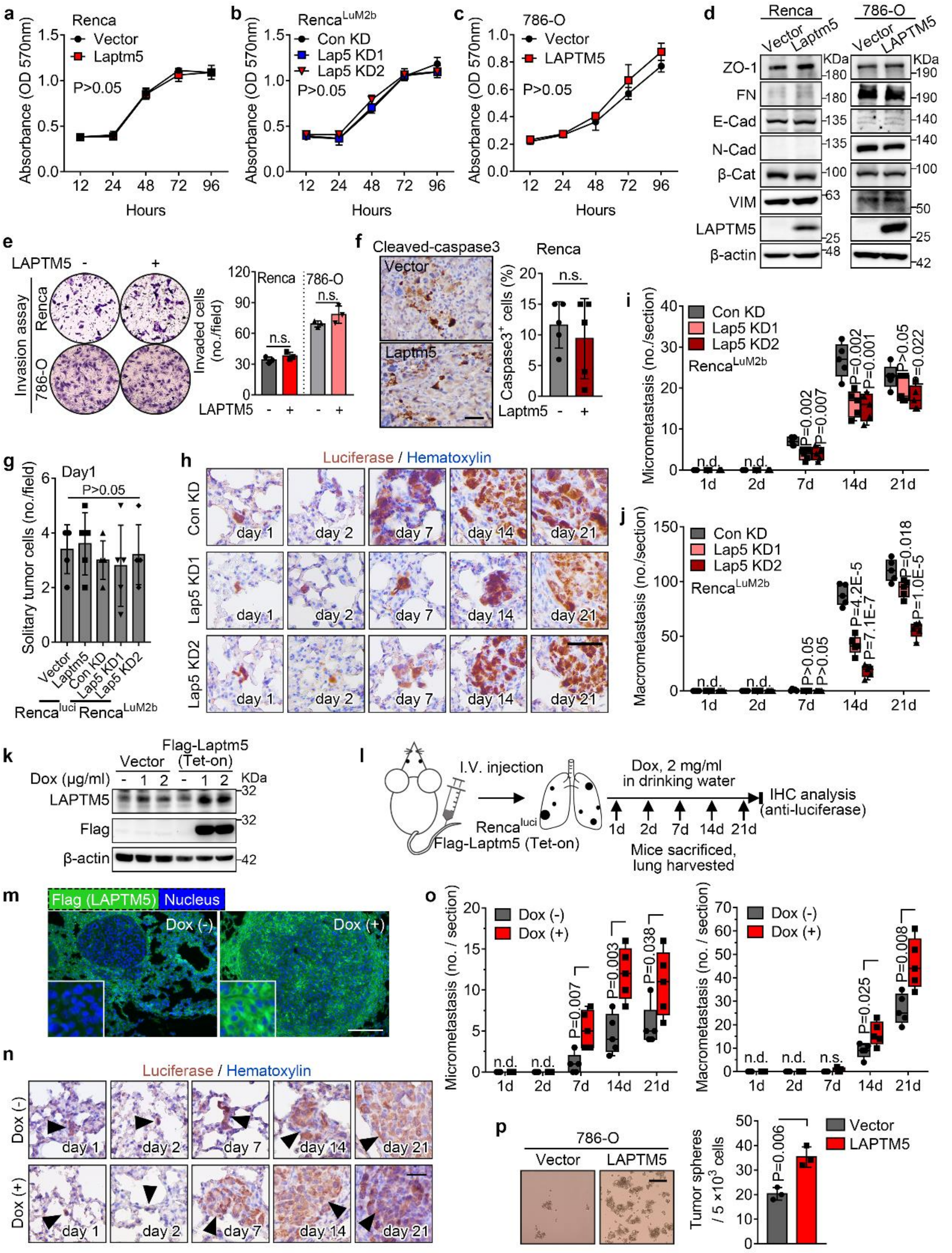

Supplementary Fig. 4 LAPTM5 promotes self-renewal and cancer stem cell traits of RCC cells

(a-c) MTT assays of control and Laptm5-overexpressing Renca cells (a, n=6 biological replicates), control and Laptm5-silenced Renca^{LuM2b} cells (b, n=4 biological replicates), control and LAPTM5-overexpressing 786-O cells (c) (n=4 biological replicates).

(d) IB analysis of EMT related proteins in control and LAPTM5-overexpressing Renca and 786-O cells.

(e) Transwell invasion assays of Renca and 786-O cells transduced with Flag-LAPTM5

or empty vector and quantification of the invaded cells per field (n=3 biological replicates).
(f) IHC analysis of cleaved-caspase3 expression in subcutaneous tumors formed by control or Laptm5-overexpressing Renca cells and quantification of the percentage of caspase3 positive (caspase3^{+}) cells (n=5 biological replicates). Scale bar, 50 μm.
(g) Quantification of solitary tumor cells per field of microscope at day 1 in lung sections harvested from mice treated as in Figure 3A (n = 5 biological replicates).
(h) Representative IHC images of luciferase staining in lung sections harvested from mice treated as in Fig. 3a. Scale bar, 50 μm.
(i and j) Quantification of micrometastasis (diameter < 100 μm, i) and macrometastasis (diameter ≥ 100 μm, j) per lung section in (h) (n=5 biological replicates).
(k) IB analysis of LAPTM5 expression in Flag-Laptm5 (Tet-on) Renca^{luci} treated with doxycycline (Dox, 1 μg/mL or 2 μg/mL) for 48h.
(l) Schematic illustration for I.V. cell inoculation, mice administration and lung metastases detection.
(m) IF analysis of lung metastases formed by Flag-Laptm5 (Tet-on) Renca^{luci} treated with or without Dox for 21 days. Scale bar, 150 μm.
(n) Representative IHC images of luciferase staining in lung sections harvested from mice treated as in (m). Scale bar, 50 μm.
(o) Quantification of micrometastasis and macrometastasis per lung section in (N) (n=5 mice per group).
(p) Tumor sphere assay (left panel) of control and LAPTM5-overexpressing 786-O cells and quantification of tumor sphere formation of indicated cells (right panel, n=3 biological replicates per group). Scale bar, 200 μm.
Immunoblots are representative of three biological replicates. In (i), (j) and (o), Data are presented as whisker plots: midline, median; box, 25–75th percentile; whisker, minimum to maximum values. Data in rest graphs are presented as the mean ± SD. Two-way ANOVA test was used for statistical analysis in (a), (b), and (c), two-tailed Student's unpaired *t*-test for (e), (f), (i), (j), (o) and (p), one-way ANOVA for (g). Source data are provided as a Source data file.

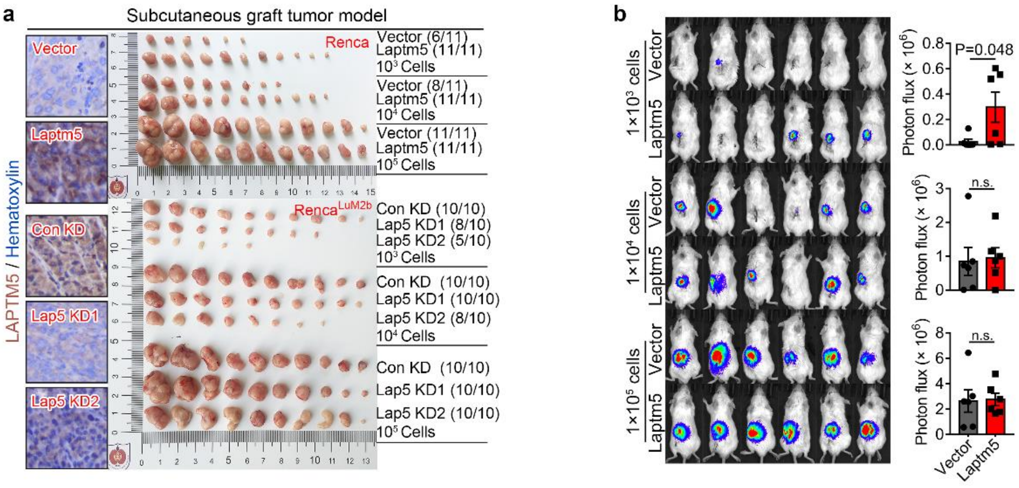

Supplementary Fig. 5 LAPTM5 promotes self-renewal and cancer stem cell traits of RCC cells

(a) IHC analysis of LAPTM5 expression in subcutaneous tumors formed by indicated cells (left panel) and image of subcutaneous tumors formed by indicated cells with various cell number (right panel). Scale bar, 50 μm.

(b) Bioluminescent images of orthotopic RCC tumors in mice implanted with Renca^{luci} and derivatives (left panel), and quantification of photon flux of orthotopic RCC tumors (right panel, n=6 mice per group). The data represent the mean ± SEM. Two-sided Student's unpaired *t* test was used for statistical analysis. Source data are provided as a Source data file.

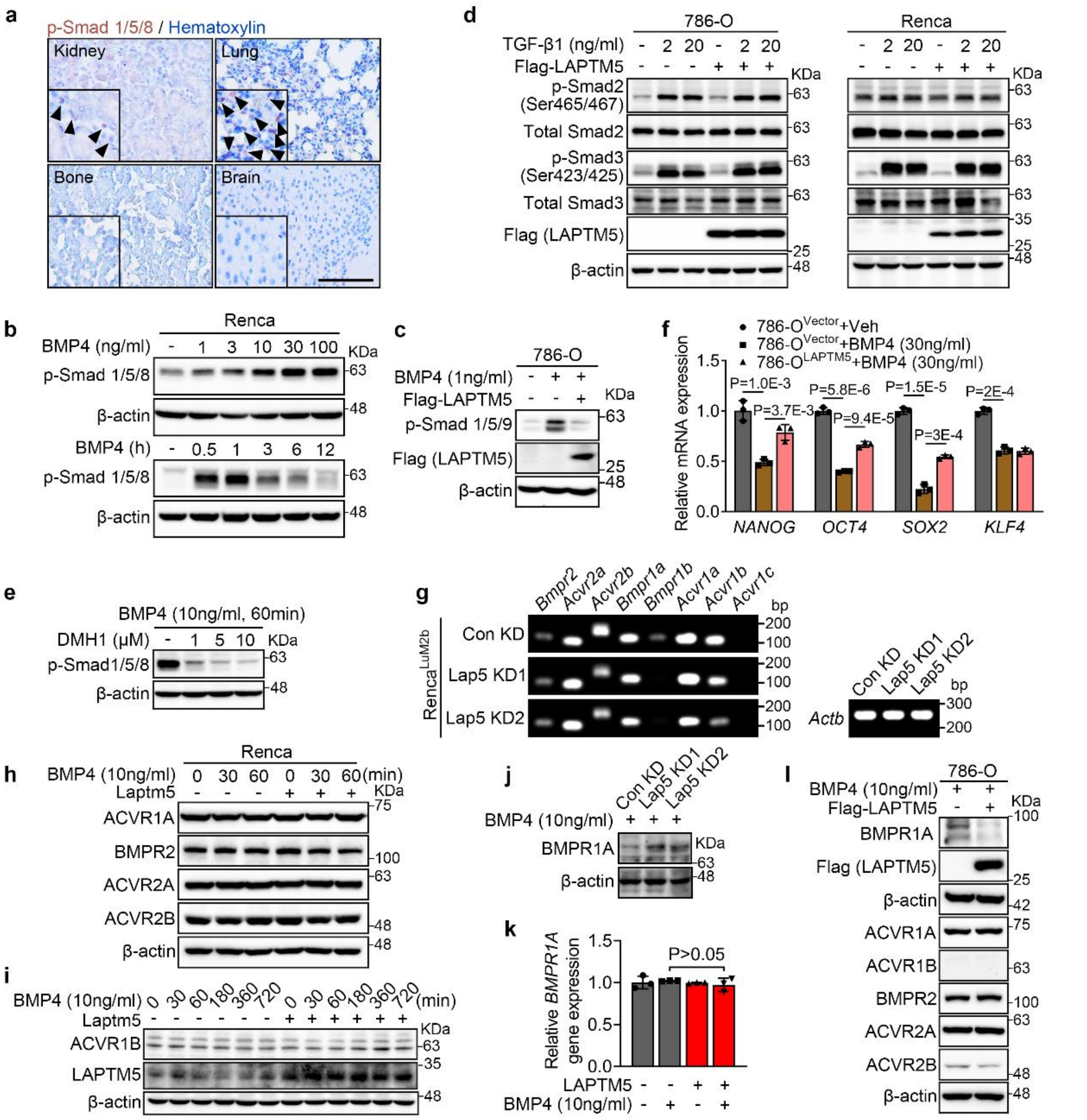

Supplementary Fig. 6 LAPTM5 blocks BMP signal and negatively regulates BMPR1A

(a) IHC analysis of p-Smad 1/5/8 staining in renal parenchymal cells and stromal cells in lung, bone and brain tissues from Renca^{luci} bearing mice. Scale bar, 100 μm.

(b) IB analysis of p-Smad 1/5/8 in Renca cells treated with different concentrations of recombinant murine BMP4 for 60 min (upper panel) or 10 ng/mL BMP4 for different times (lower panel).

(c) IB analysis of p-Smad 1/5/9 in control and LAPTM5-overexpressing 786-O cells treated with BMP4 (1 ng/mL) for 60 min.

(d) IB analysis of control and LAPTM5-overexpressing 786-O and Renca cells treated with recombinant TGF-β1 (2 ng/mL or 20 ng/mL) for 60 min.

(e) IB analysis of p-Smad 1/5/8 in Renca cells treated with BMP4 (10 ng/mL) and/or different concentrations of DMH1 for 60 min.

(f) qRT-PCR analysis of stemness markers in indicated cells. n=3 biological replicates.

(g) Semiquantitative RT-PCR analysis of BMP receptors in control and Laptm5-silenced Renca^{LuM2b} cells with *Actb* as control.

(h and i) IB analysis of BMP receptors in control and Laptm5-overexpressing Renca cells treated with BMP4 (10 ng/mL) for indicated times.
(j) IB analysis of BMPR1A in control and Laptm5-silenced Renca^{LuM2b} cells treated with BMP4 (10 ng/mL) for 60 min.
(k) qRT-PCR analysis of *BMPR1A* in control and LAPTM5-overexpressing 786-O cells treated with BMP4 (10 ng/mL) for 60 min. n=3 biological replicates.
(l) IB analysis of BMP receptors in control and LAPTM5-overexpressing 786-O cells treated with BMP4 (10 ng/mL) for 60 min.
Immunoblots are representative of three biological replicates. The data in bar graphs are presented as mean ± SD. Two-tailed Student's unpaired *t*-test was used for statistical analysis in all panels. Source data are provided as a Source data file.

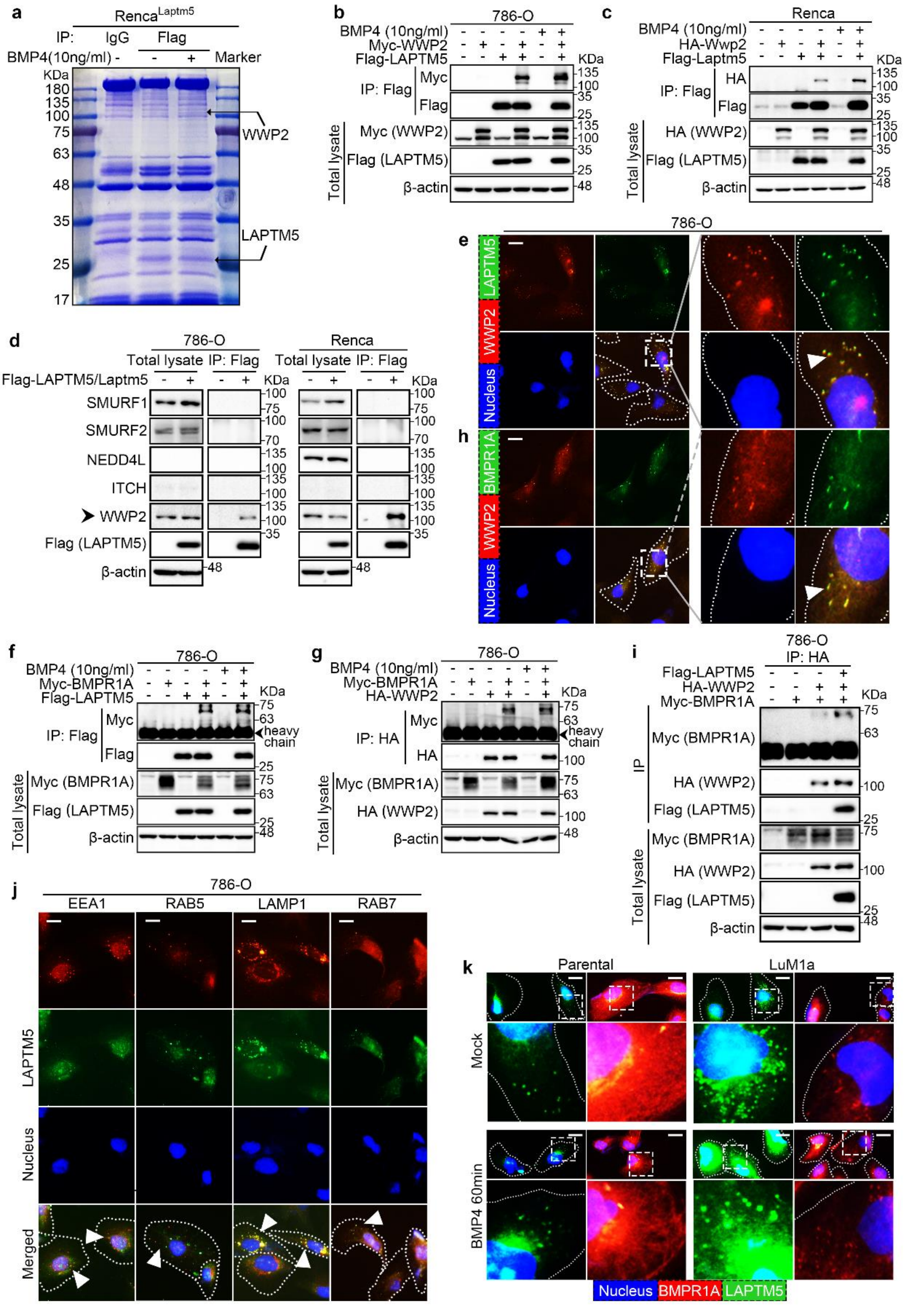

Supplementary Fig. 7 LAPTM5 directly interact with WWP2 and BMPR1A

(a) Coomassie brilliant blue staining of RencaLaptm5 cells treated or untreated with BMP4 (10 ng/mL) for 60 min. The arrowheads indicate bands of WWP2 and LAPTM5, respectively.

(b and c) Immunoprecipitation (IP) and IB analyses of 786-O (b) and Renca (c) cells

transfected with expression vectors for Flag-LAPTM5, Myc-WWP2 or HA-Wwp2, and treated with BMP4 (10 ng/mL) for 60 min.

(d) IP and IB analyses of NEDD4 family proteins in 786-O (left panel) and Renca (right panel) cells transfected with expression vectors for Flag-LAPTM5.

(e) IF analysis of 786-O cells co-stained for LAPTM5 (green) and WWP2 (red). Scale bar, 10 μm.

(f and g) IP and IB analyses of 786-O cells transfected with expression vectors for Myc-BMPR1A and Flag-LAPTM5 (f) or HA-WWP2 (g) and treated with BMP4 (10 ng/mL) for 60 min.

(h) IF analysis of 786-O cells co-stained for BMPR1A (green) and WWP2 (red). Scale bar, 10 μm.

(i) IP and IB analyses of 786-O cells transfected with expression vectors for Flag-LAPTM5, HA-WWP2, and Myc-BMPR1A.

(j) IF analysis of 786-O cells co-stained for LAPTM5 (green) and early endosome markers (EEA1 and RAB5, red) or late endosome markers (LAMP1 and RAB7, red). Scale bar, 10 μm.

(k) IF analysis of $786O^{luci/eGFP}$ parental and LuM1a cells for BMPR1A (red) and LAPTM5 (green). Scale bar, 10 μm.

Immunoblots are representative of three biological replicates.

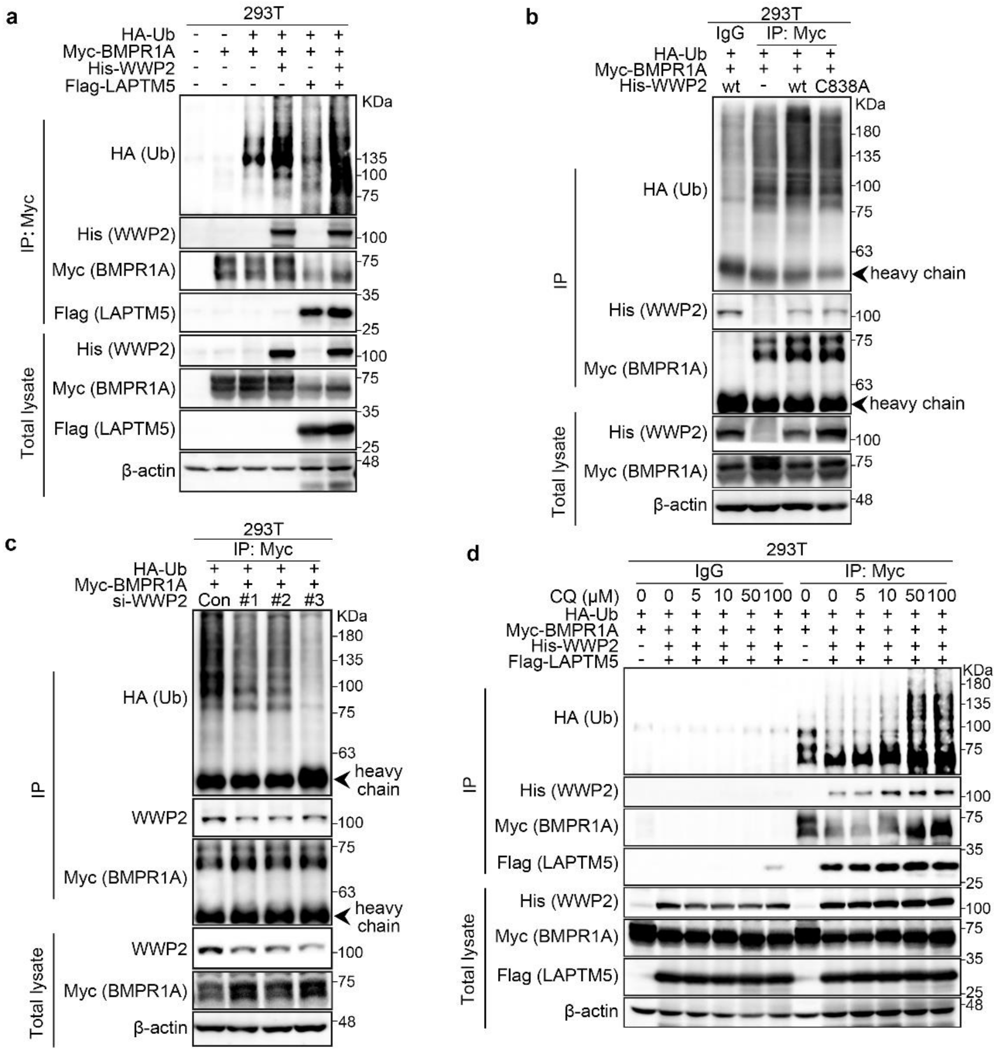

Supplementary Fig. 8 WWP2 promotes lysosome-based polyubiquitylation and degradation of BMPR1A

(a) IP and IB analyses of 293T cells transfected with expression vectors for Flag-LAPTM5, His-WWP2, Myc-BMPR1A and HA-ubiquitin (Ub).

(b) IP and IB analyses of 293T cells transfected with expression vectors for His-tagged wild type (wt) or C838A mutated WWP2, Myc-BMPR1A and HA-Ub.

(c) IP and IB analyses of 293T cells transfected with siRNAs against WWP2 and expression vectors for Myc-BMPR1A and HA-Ub.

(d) IP and IB analyses of 293T cells transfected with expression vectors for Flag-LAPTM5, His-WWP2, Myc-BMPR1A, HA-Ub and treated with different concentrations of chloroquine (CQ) for 60 min.

Immunoblots are representative of three biological replicates.

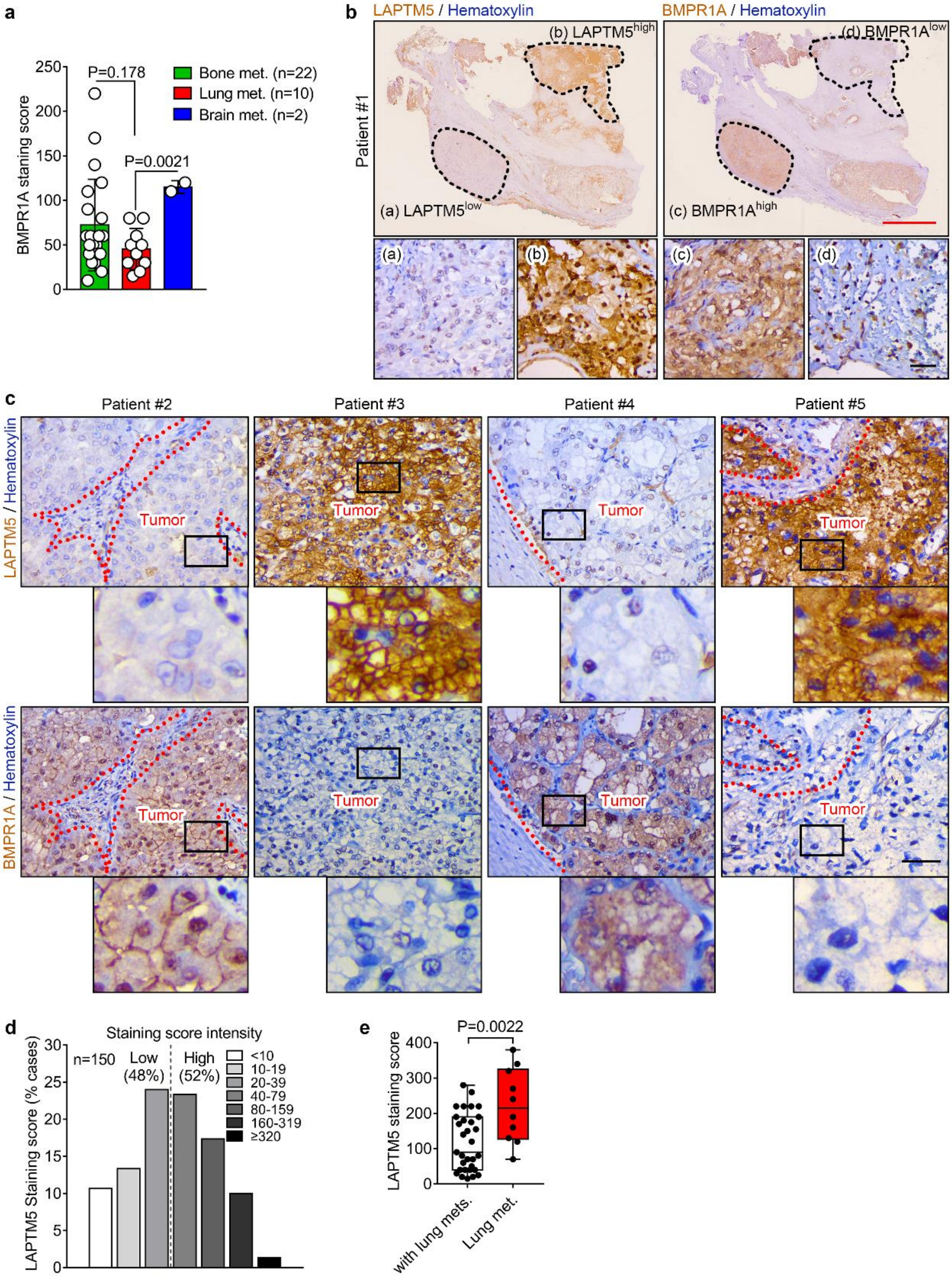

Supplementary Fig. 9 LAPTM5 negatively correlates with BMPR1A and predicts lung metastasis of RCC

(a) IHC staining score of BMPR1A in clinical organ metastases from RCC. The data represent the mean ± SEM.

(b) Representative IHC images for LAPTM5 (left panel) and BMPR1A (right panel) in serial sections of clinical primary RCC tissue. The whole section scanning images showed the LAPTM5^{low} (a) versus LAPTM5^{high} (b) and BMPR1A^{high} (c) versus

BMPR1A^{low} (d) zones. Red scale bar, 5 mm; black scale bar, 50 μm.
(c) Representative IHC images for LAPTM5 (upper panel) and BMPR1A (lower panel) in serial sections of clinical primary RCC tissues. Scale bar, 10 μm.
(d) Distribution of LAPTM5 staining score across clinical primary RCC tissues.
(E) Staining score of LAPTM5 in primary RCC tissue with lung metastases (with lung met., n=31) and the lung metastases (n=10). Data are presented as whisker plots: midline, median; box, 25–75th percentile; whisker, minimum to maximum values.
Two-tailed Student's unpaired *t*-test was used for statistical analysis in all panels. Source data are provided as a Source data file.

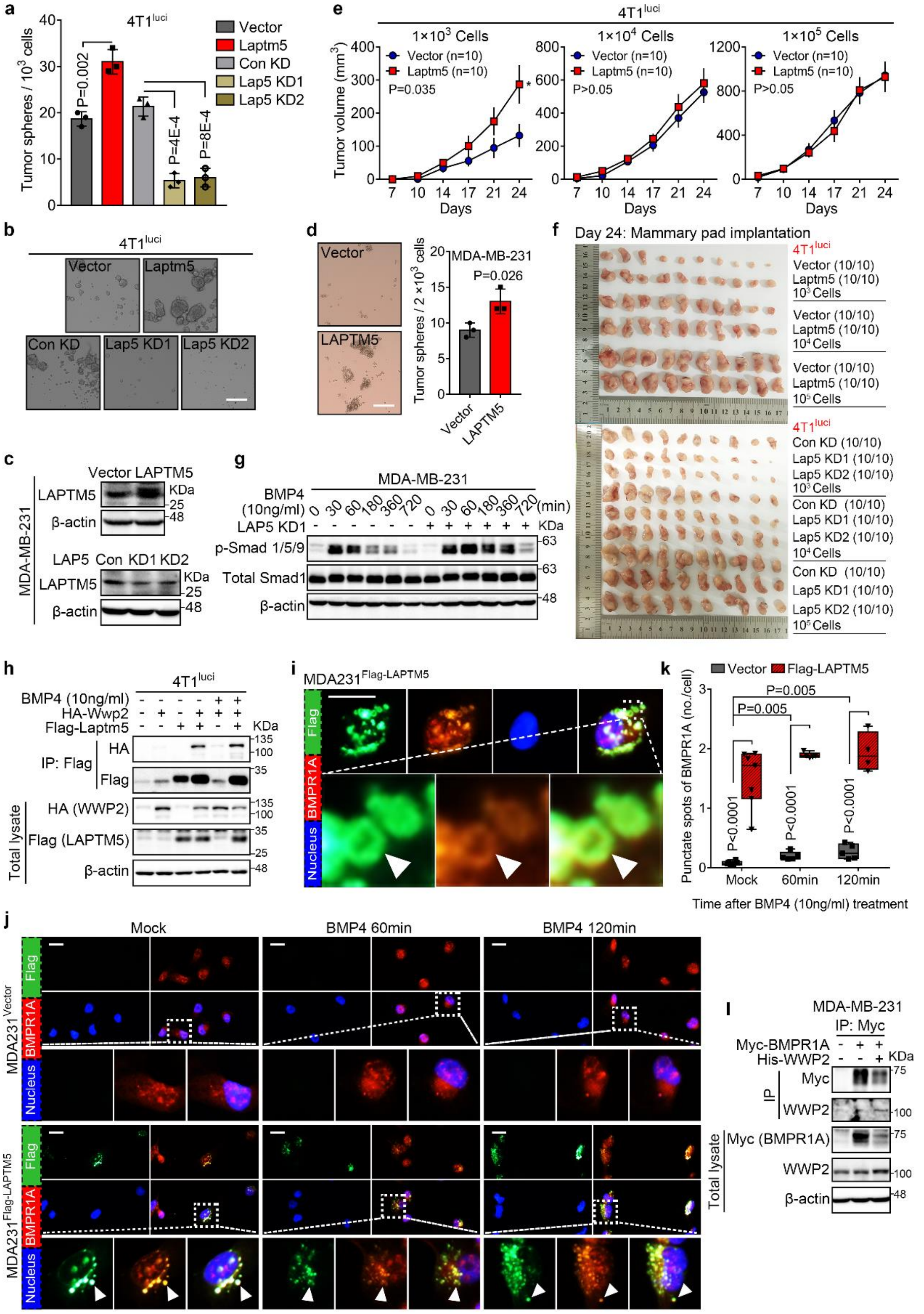

Supplementary Fig. 10 LAPTM5 is specifically activated in lung metastases of multiple cancers

(a and b) Quantification (a) and representative images (b) of tumor sphere assay (n=3 biological replicates) in the murine breast cancer cell line 4T1. Scale bar, 200 μm.

(c) IB analysis of LAPTM5 in control and LAPTM5-overexpressing and control and

LAPTM5-silenced MDA-MB-231 cells.
(d) Tumor sphere assay (left panel) and quantification of tumor sphere formation of control and LAPTM5-overexpressing MDA-MB-231 cells (n=3 biological replicates). Scale bar, 200 μm.
(e) Tumor volumes of control and Laptm5-overexpressing $4T1^{luci}$ cells inoculated *in situ* as described in Figure 8F. n=10 mice per group. The data represent the mean ± SEM.
(f) Image of orthotopic tumors formed by indicated cells with different cell number in (e).
(g) IB analysis of p-smad 1/5/9 in control and LAPTM5-silenced MDA-MB-231 cells treated with BMP4 (10 ng/mL) for respective durations.
(h) IP and IB analyses of $4T1^{luci}$ cells transfected with expression vectors for Flag-Laptm5, HA-Wwp2, and treated with BMP4 (10 ng/mL) for 60 min.
(i) IF analysis of LAPTM5-overexpressing MDA-MB-231 cells co-stained for LAPTM5 (green) and BMPR1A (red). Scale bar, 10 μm.
(j) IF analysis of control and LAPTM5-overexpressing MDA-MB-231 cells treated with Mock (ddH_2O), or BMP4 (10 ng/mL) for 60 min or 120 min. Scale bar, 10 μm.
(k) Quantification of BMPR1A punctate spots per MDA-MB-231 cell treated as in (J) [n = biological replicates, 6 in Vector, 7 in LAPTM5 (Mock); n=5 in Vector, 5 in LAPTM5 (60 min); n=5 in Vector, 4 in LAPTM5 (120 min)]. Data are presented as whisker plots: midline, median; box, 25–75th percentile; whisker, minimum to maximum values.
(l) IP and IB analyses of MDA-MB-231 cells transfected with expression vectors for Myc-BMPR1A and His-WWP2.
Immunoblots are representative of three biological replicates. In (a), (d), and (k), the data are presented as mean ± SD. Two-tailed Student's unpaired *t*-test was used for statistical analysis in all panels. Source data are provided as a Source data file.
