# Supplementary material for: Lysosomal protein transmembrane 5 promotes lung-specific metastasis by regulating BMPR1A lysosomal degradation
Source: Nat Commun. 2022 Jul 16;13:4141. doi: 10.1038/s41467-022-31783-6 (PMC9288479; doi:10.1038/s41467-022-31783-6)
Supplement: Supplementary file 3 — Description of Additional Supplementary Files [file 41467_2022_31783_MOESM3_ESM.pdf]

Description of Additional Supplementary Files

Supplementary Data 1
69 up-regulated genes identified with lung metastatic phenotypes in the multi-organ metastasis model.

Supplementary Data 2
3 up-regulated genes identified with lung metastatic phenotypes in both the multi-organ metastasis model and the Jon_Renal_Cancer datasets.

Supplementary Data 3
GSEA result with LAPTM5 expression in the TCGA KIRC cohort.

Supplementary Data 4
GSEA result with LAPTM5 expression in the TCGA KIRP cohort.

Supplementary Data 5
Correlation of LAPTM5 expression to clinicopathological features in 150 KIRC patients.

Supplementary Data 6
Nucleotide sequences of the primers used in the PCR assays.

Supplementary Data 7
Information on relevant reagents and materials used in this study.
