# Supplementary material for: Lysosomal protein transmembrane 5 promotes lung-specific metastasis by regulating BMPR1A lysosomal degradation
Source: Nat Commun. 2022 Jul 16;13:4141. doi: 10.1038/s41467-022-31783-6 (PMC9288479; doi:10.1038/s41467-022-31783-6)
Supplement: Supplementary file 11 — Reporting Summary [file 41467_2022_31783_MOESM11_ESM.pdf]

nature portfolio

Corresponding author(s): Hongqian Guo

Last updated by author(s): Jun 1, 2022

Reporting Summary

Nature Portfolio wishes to improve the reproducibility of the work that we publish. This form provides structure for consistency and transparency in reporting. For further information on Nature Portfolio policies, see our Editorial Policies and the Editorial Policy Checklist.

Statistics

For all statistical analyses, confirm that the following items are present in the figure legend, table legend, main text, or Methods section.

n/a	Confirmed	
☐	☒	The exact sample size (*n*) for each experimental group/condition, given as a discrete number and unit of measurement
☐	☒	A statement on whether measurements were taken from distinct samples or whether the same sample was measured repeatedly
☐	☒	The statistical test(s) used AND whether they are one- or two-sided<br>*Only common tests should be described solely by name; describe more complex techniques in the Methods section.*
☒	☐	A description of all covariates tested
☒	☐	A description of any assumptions or corrections, such as tests of normality and adjustment for multiple comparisons
☐	☒	A full description of the statistical parameters including central tendency (e.g. means) or other basic estimates (e.g. regression coefficient) AND variation (e.g. standard deviation) or associated estimates of uncertainty (e.g. confidence intervals)
☐	☒	For null hypothesis testing, the test statistic (e.g. *F*, *t*, *r*) with confidence intervals, effect sizes, degrees of freedom and *P* value noted<br>*Give P values as exact values whenever suitable.*
☒	☐	For Bayesian analysis, information on the choice of priors and Markov chain Monte Carlo settings
☒	☐	For hierarchical and complex designs, identification of the appropriate level for tests and full reporting of outcomes
☐	☒	Estimates of effect sizes (e.g. Cohen's *d*, Pearson's *r*), indicating how they were calculated

Our web collection on statistics for biologists contains articles on many of the points above.

Software and code

Policy information about availability of computer code

Data collection	RNA-seq data were collected with Illumina Novaseq platform. BLI data was acquired using IVIS Spectrum Imaging System (Caliper Life Sciences) and Living Image software v 4.5.5. qPCR data were acquired with QuantStudio 6 Flex Software v 1.3. Mass cytometry data were collected with Thermo Q Exactive and software Proteome Discoverer 2.1. Immunoblotting data were acquired using CLiNX platform. Histology data for H&E and IHC were acquired using Leica Microsystems and Leica Application Suite v 4.12.0. Immunofluorescence and cell morphology data were collected using EVOS Auto 2 (Invitrogen) and EVOS FL Auto 2.0 Imaging System. MTT data were acquired using Tecan i-control.
Data analysis	BLI data were analyzed using Living Image software v 4.5.5. Gene Set Enrichment Analysis (GSEA) was analyzed by pre-Ranked GSEA (preRankedGSEA) on genes ranked by Spearman correlation coefficient. The correlation coefficients were sorted in descending order and submitted to “GSEA” function of R “clusterProfiler” package with default parameters. The gene sets were obtained from the Molecular Signatures Database (MSigDB 7.1) and the pathways curated from the Pathway Interaction Database (PID) were used in this analysis. The GSEA results were further visualized by R “enrichplot” package. The quantification of immunoblottings were using ImageJ (v 1.53). Figure 3G, 7F and Table S5 were analyzed with SPSS Statistics 21.0. All other data were analyzed with Graphpad Prism 8 for Windows.

For manuscripts utilizing custom algorithms or software that are central to the research but not yet described in published literature, software must be made available to editors and reviewers. We strongly encourage code deposition in a community repository (e.g. GitHub). See the Nature Portfolio guidelines for submitting code & software for further information.

Data

Policy information about availability of data

All manuscripts must include a data availability statement. This statement should provide the following information, where applicable:

- Accession codes, unique identifiers, or web links for publicly available datasets
- A description of any restrictions on data availability
- For clinical datasets or third party data, please ensure that the statement adheres to our policy

The RNA-seq data have been deposited in the National Center for Biotechnology Information Sequence Read Archive (SRA) database (https://www.ncbi.nlm.nih.gov/sra/PRJNA724865). Processed data of breast cancer (accession number: GSE2603), melanoma (accession number: GSE50496) and colorectal cancer (accession number: GSE41258) were collected from Gene Expression Omnibus (GEO) database. Processed data of Jon_Renal_Cancer were collected from supplementary information of this paper (DOI: 10.1158/1078-0432.CCR-04-2225). All the other data supporting the findings of this study are available within the article and its Supplementary Information files. A reporting summary for this article is available as a Supplementary Information file. Source data are provided with this paper.

Human research participants

Policy information about studies involving human research participants and Sex and Gender in Research.

Reporting on sex and gender	The findings of this work were independent of sex. Sex was not considered in study design. A total of 184 patients and 290 samples were included in the study. Sex-based analysis was performed in Fig. 7f.
Population characteristics	Formalin-fixed RCC metastases from various organs (10 lung metastases, 22 bone metastases, 2 brain metastases), primary RCC tissues (n=150) and noncancerous kidney tissues (n=106) were collected from patients undergoing biopsies or surgical resection from 2005 to 2019. Cancerous tissue was classified according to the WHO classification at the Nanjing University Medical School affiliated Nanjing Drum Tower Hospital.
Recruitment	We randomly selected 184 patients with complete basic information. There is no potential self-selection bias or other biases.
Ethics oversight	Ethics approval was obtained from the Nanjing University Medical School affiliated Nanjing Drum Tower Hospital.

Note that full information on the approval of the study protocol must also be provided in the manuscript.

Field-specific reporting

Please select the one below that is the best fit for your research. If you are not sure, read the appropriate sections before making your selection.

☒ Life sciences ☐ Behavioural & social sciences ☐ Ecological, evolutionary & environmental sciences

For a reference copy of the document with all sections, see nature.com/documents/nr-reporting-summary-flat.pdf

Life sciences study design

All studies must disclose on these points even when the disclosure is negative.

Sample size	Sample size for each experiment are stated in figure legends. For in vitro assays, n = 3-9 / group. For in vivo mouse experiments, n=5-11 mice were used for analyses. No statistical method was used to predetermine sample size. Sample sizes are determined empirically, and similar in size to most existing studies in the same field (PMID: 22901808; PMID: 27982029; PMID: 32094692; PMID: 27368100).
Data exclusions	No data were excluded.
Replication	Biological replicates of each experiment is stated under each figure legend and all attempts were successful.
Randomization	For all in vivo experiments, mice were randomly allocated among groups. For IHC and IF assays, 5 fields of view were randomly selected for each slice.
Blinding	RNA-seq data analysis was double-blinded, and hierarchical clustering indicated significant differences and correct grouping before sample identity disclosure. For animal experiments, investigators were not blinded of group assignment during data collection and/or analysis since because gave different treatments between groups (PMID: 22901808; PMID: 24529372; PMID: 26603525). Since the mice were randomly allocated among groups, blinding should not be relevant in these analyses. The experimental results of molecular and cellular biology are obtained by objective quantitative methods (PMID: 22901808), this precludes effectively blinding.

Reporting for specific materials, systems and methods

We require information from authors about some types of materials, experimental systems and methods used in many studies. Here, indicate whether each material, system or method listed is relevant to your study. If you are not sure if a list item applies to your research, read the appropriate section before selecting a response.

Materials & experimental systems

n/a	Involved in the study
☐	☒ Antibodies
☐	☒ Eukaryotic cell lines
☒	☐ Palaeontology and archaeology
☐	☒ Animals and other organisms
☒	☐ Clinical data
☒	☐ Dual use research of concern

Methods

n/a	Involved in the study
☒	☐ ChIP-seq
☒	☐ Flow cytometry
☒	☐ MRI-based neuroimaging

Antibodies

Antibodies used

Rabbit anti-mouse LAPTM5 Vazyme Biotech N/A
Rabbit anti-LAPTM5 Abcam Cat# ab108014
Mouse anti-β-actin Proteintech Cat# 66009-1-Ig
Mouse anti-Luciferase Santa Cruz Biotechnology Cat# sc-74548
Rabbit anti-cleaved Caspase-3 Cell Signaling Technology Cat# 9661
Rabbit anti-ZO-1 Proteintech Cat# 21773-1-AP
Rabbit anti-Fibronectin Abcam Cat# ab2413
Rabbit anti-E-cadherin Proteintech Cat# 20874-1-AP
Rabbit anti-N-cadherin Proteintech Cat# 22018-1-AP
Rabbit anti-β-catanin Proteintech Cat# 51067-2-AP
Rabbit anti-Vimentin Proteintech Cat# 10366-1-AP
Rabbit anti-phospho-SMAD1/5/9 Cell Signaling Technology Cat# 13820
Rabbit anti-SMAD1 Cell Signaling Technology Cat# 6944
Rabbit anti-phospho-SMAD2 Cell Signaling Technology Cat# 3108
Rabbit anti-SMAD2 Cell Signaling Technology Cat# 5339
Rabbit anti-phospho-SMAD3 Abcam Cat# ab52903
Rabbit anti-SMAD3 Cell Signaling Technology Cat# 9523
Rabbit anti-BMPR1A Thermo Fisher Scientific Cat# 38-6000
Mouse anti-BMPR1A Santa Cruz Biotechnology Cat# sc-518037
Mouse anti-ACVR1A Santa Cruz Biotechnology Cat# sc-374523
Mouse anti-ACVR1B Santa Cruz Biotechnology Cat# sc-73677
Mouse anti-BMPR2 Santa Cruz Biotechnology Cat# sc-393304
Mouse anti-ACVR2A Santa Cruz Biotechnology Cat# sc-515826
Mouse anti-ACVR2B Santa Cruz Biotechnology Cat# sc-376593
Mouse anti-Flag-tag Sigma-Aldrich Cat# F1804
Rabbit anti-Flag-tag Cell Signaling Technology Cat# 14793
Rabbit anti-HA-tag Cell Signaling Technology Cat# 3724
Rabbit anti-Myc-tag Abcam Cat# ab9106
Mouse anti-His-tag Bioworld Technology Cat# AP0032M
Mouse anti-WWP2 Santa Cruz Biotechnology Cat# sc-398090
Mouse anti-SMURF1 Santa Cruz Biotechnology Cat# sc-100616
Rabbit anti-SMURF2 Abcam Cat# ab53316
Mouse anti-NEDD4L Santa Cruz Biotechnology Cat# sc-514954
Mouse anti-ITCH Santa Cruz Biotechnology Cat# sc-28367
Mouse anti-Rab5 Santa Cruz Biotechnology Cat# sc-46692
Mouse anti-Rab7 Santa Cruz Biotechnology Cat# sc-376362
Mouse anti-EEA1 Santa Cruz Biotechnology Cat# sc-365652
Mouse anti-LAMP1 Santa Cruz Biotechnology Cat# sc-20011
Normal rabbit IgG Cell Signaling Technology Cat# 2729
Goat anti-Rabbit IgG (H+L) Highly Cross-Adsorbed Secondary Antibody, Alexa Fluor 594 Thermo Fisher Scientific Cat# A11037
Goat anti-Mouse IgG (H+L) Highly Cross-Adsorbed Secondary Antibody, Alexa Fluor 488 Thermo Fisher Scientific Cat# A11029
Goat anti-Mouse IgG (H+L) Cross-Adsorbed Secondary Antibody, Alexa Fluor 594 Thermo Fisher Scientific Cat# A11005
Goat anti-Rabbit IgG (H+L) Highly Cross-Adsorbed Secondary Antibody, Alexa Fluor 488 Thermo Fisher Scientific Cat# A11034
Peroxidase labeled anti-Rabbit IgG (H+L), made in goat "Vector Laboratories, Inc." Cat# PI-1000
Peroxidase labeled anti-Mouse IgG (H+L), made in horse "Vector Laboratories, Inc." Cat# PI-2000

Validation

Polyclonal rabbit antibodies were raised against a peptide (PPKTPEGDPAPPPYSEV) located near the C terminus of mouse LAPTM5 (PMIDall other commercial primary antibodies: 18619870). The rabbit anti-mouse LAPTM5 antibody was produced by Vazyme Biotech and was validated with mouse recombinant Flag-tagged LAPTM5 fusion protein in mouse Renca cells in WB and IHC assays. Rabbit anti-LAPTM5 Abcam Cat# ab108014 was validated with human recombinant Flag-tagged LAPTM5 fusion protein in human 786-O cells in IF assay.

Validation of all other commercial primary antibodies was relied on the available data on commercial websites.
The following antibodies have been approved by the manufacturers for WB assay:
Rabbit anti-LAPTM5 Abcam Cat# ab108014/https://www.abcam.cn/laptm5-antibody-ab108014.html
Mouse anti-β-actin Proteintech Cat# 66009-1-Ig/https://www.ptglab.com/products/Pan-Actin-Antibody-66009-1-Ig.htm
Rabbit anti-ZO-1 Proteintech Cat# 21773-1-AP/https://www.ptglab.com/products/ZO1-Antibody-21773-1-AP.htm
Rabbit anti-Fibronectin Abcam Cat# ab2413/https://www.abcam.cn/fibronectin-antibody-ab2413.html
Rabbit anti-E-cadherin Proteintech Cat# 20874-1-AP/https://www.ptglab.com/products/E-cadherin-Antibody-20874-1-AP.htm
Rabbit anti-N-cadherin Proteintech Cat# 22018-1-AP/https://www.ptglab.com/products/N-cadherin-Antibody-22018-1-AP.htm
Rabbit anti-β-catanin Proteintech Cat# 51067-2-AP/https://www.ptglab.com/products/b-cat-Antibody-51067-2-AP.htm
Rabbit anti-Vimentin Proteintech Cat# 10366-1-AP/https://www.ptglab.com/products/VIM-Antibody-10366-1-AP.htm
Rabbit anti-phospho-SMAD1/5/9 Cell Signaling Technology Cat# 13820/https://www.cellsignal.cn/products/primary-antibodies/phospho-smad1-ser463-465-smad5-ser463-465-smad9-ser465-467-d5b10-rabbit-mab/13820?site-search-type=Products&N=4294956287&Ntt=13820&fromPage=plp&_requestid=3445476
Rabbit anti-SMAD1 Cell Signaling Technology Cat# 6944/https://www.cellsignal.cn/products/primary-antibodies/smad1-d59d7-xp-rabbit-mab/6944?site-search-type=Products&N=4294956287&Ntt=6944&fromPage=plp&_requestid=3445773
Rabbit anti-phospho-SMAD2 Cell Signaling Technology Cat# 3108/https://www.cellsignal.cn/products/primary-antibodies/phospho-smad2-ser465-467-138d4-rabbit-mab/3108?site-search-type=Products&N=4294956287&Ntt=3108&fromPage=plp&_requestid=3445805
Rabbit anti-SMAD2 Cell Signaling Technology Cat# 5339/https://www.cellsignal.cn/products/primary-antibodies/smad2-d43b4-xp-rabbit-mab/5339?site-search-type=Products&N=4294956287&Ntt=5339&fromPage=plp&_requestid=3445878
Rabbit anti-phospho-SMAD3 Abcam Cat# ab52903/https://www.abcam.cn/smad3-phospho-s423--s425-antibody-ep823y-ab52903.html
Rabbit anti-SMAD3 Cell Signaling Technology Cat# 9523/https://www.cellsignal.cn/products/primary-antibodies/smad3-c67h9-rabbit-mab/9523?site-search-type=Products&N=4294956287&Ntt=9523&fromPage=plp&_requestid=3445908
Mouse anti-BMPR1A Santa Cruz Biotechnology Cat# sc-518037/https://www.scbt.com/p/bmpr-ia-antibody-c-7?requestFrom=search
Mouse anti-ACVR1A Santa Cruz Biotechnology Cat# sc-374523/https://www.scbt.com/p/actr-i-antibody-c-5?requestFrom=search
Mouse anti-ACVR1B Santa Cruz Biotechnology Cat# sc-73677/https://www.scbt.com/p/actr-ib-antibody-alex66?requestFrom=search
Mouse anti-BMPR2 Santa Cruz Biotechnology Cat# sc-393304/https://www.scbt.com/p/bmpr-ii-antibody-e-1?requestFrom=search
Mouse anti-ACVR2A Santa Cruz Biotechnology Cat# sc-515826/https://www.scbt.com/p/actr-iia-antibody-d-9?requestFrom=search
Mouse anti-ACVR2B Santa Cruz Biotechnology Cat# sc-376593/https://www.scbt.com/p/actr-iib-antibody-g-7?requestFrom=search
Mouse anti-Flag-tag Sigma-Aldrich Cat# F1804/https://www.sigmaaldrich.cn/CN/zh/product/sigma/f1804
Rabbit anti-Flag-tag Cell Signaling Technology Cat# 14793/https://www.cellsignal.cn/products/primary-antibodies/dykddddk-tag-d6w5b-rabbit-mab-binds-to-same-epitope-as-sigma-s-anti-flag-m2-antibody/14793?site-search-type=Products&N=4294956287&Ntt=14793&fromPage=plp&_requestid=3446810
Rabbit anti-HA-tag Cell Signaling Technology Cat# 3724/https://www.cellsignal.cn/products/primary-antibodies/ha-tag-c29f4-rabbit-mab/3724?site-search-type=Products&N=4294956287&Ntt=3724&fromPage=plp&_requestid=3447125
Rabbit anti-Myc-tag Abcam Cat# ab9106/https://www.abcam.cn/myc-tag-antibody-ab9106.html
Mouse anti-His-tag Bioworld Technology Cat# AP0032M/https://www.bioworlde.com/Primary-Antibodies/45399.html
Mouse anti-WWP2 Santa Cruz Biotechnology Cat# sc-398090/https://www.scbt.com/p/aip2-antibody-a-3?requestFrom=search
Mouse anti-SMURF1 Santa Cruz Biotechnology Cat# sc-100616/https://www.scbt.com/p/smurf1-antibody-45-k?requestFrom=search
Rabbit anti-SMURF2 Abcam Cat# ab53316/https://www.abcam.cn/smurf-2-antibody-ep629y3-ab53316.html
Mouse anti-NEDD4L Santa Cruz Biotechnology Cat# sc-514954/https://www.scbt.com/p/nedd4-l-antibody-c-8?requestFrom=search
Mouse anti-ITCH Santa Cruz Biotechnology Cat# sc-28367/https://www.scbt.com/p/aip4-antibody-g-11?requestFrom=search

The following antibodies have been approved by the manufacturers for IP assay:
Rabbit anti-Flag-tag Cell Signaling Technology Cat# 14793/https://www.cellsignal.cn/products/primary-antibodies/dykddddk-tag-d6w5b-rabbit-mab-binds-to-same-epitope-as-sigma-s-anti-flag-m2-antibody/14793?site-search-type=Products&N=4294956287&Ntt=14793&fromPage=plp&_requestid=3446810
Rabbit anti-HA-tag Cell Signaling Technology Cat# 3724/https://www.cellsignal.cn/products/primary-antibodies/ha-tag-c29f4-rabbit-mab/3724?site-search-type=Products&N=4294956287&Ntt=3724&fromPage=plp&_requestid=3447125
Rabbit anti-Myc-tag Abcam Cat# ab9106/https://www.abcam.cn/myc-tag-antibody-ab9106.html
Mouse anti-His-tag Bioworld Technology Cat# AP0032M/https://www.bioworlde.com/Primary-Antibodies/45399.html
Mouse anti-WWP2 Santa Cruz Biotechnology Cat# sc-398090/https://www.scbt.com/p/aip2-antibody-a-3?requestFrom=search
Normal rabbit IgG Cell Signaling Technology Cat# 2729/https://www.cellsignal.cn/products/primary-antibodies/normal-rabbit-igg/2729?site-search-type=Products&N=4294956287&Ntt=2729&fromPage=plp&_requestid=3448263

The following antibodies have been approved by the manufacturers for IHC assay:
Rabbit anti-LAPTM5 Abcam Cat# ab108014/https://www.abcam.cn/laptm5-antibody-ab108014.html
Mouse anti-Luciferase Santa Cruz Biotechnology Cat# sc-74548/https://www.scbt.com/p/luciferase-antibody-c-12?requestFrom=search
Rabbit anti-phospho-SMAD1/5/9 Cell Signaling Technology Cat# 13820/https://www.cellsignal.cn/products/primary-antibodies/phospho-smad1-ser463-465-smad5-ser463-465-smad9-ser465-467-d5b10-rabbit-mab/13820?site-search-type=Products&N=4294956287&Ntt=13820&fromPage=plp&_requestid=3445476
Rabbit anti-BMPR1A Thermo Fisher Scientific Cat# 38-6000/https://www.thermofisher.cn/cn/zh/antibody/product/BMPR1A-Antibody-Polyclonal/38-6000
Rabbit anti-cleaved Caspase-3 Cell Signaling Technology Cat# 9661/https://www.cellsignal.cn/products/primary-antibodies/cleaved-caspase-3-asp175-antibody/9661?site-search-type=Products&N=4294956287&Ntt=9661&fromPage=plp&_requestid=3448390

The following antibodies have been approved by the manufacturers for IF assay:
Rabbit anti-BMPR1A Thermo Fisher Scientific Cat# 38-6000/https://www.thermofisher.cn/cn/zh/antibody/product/BMPR1A-Antibody-Polyclonal/38-6000
Mouse anti-Flag-tag Sigma-Aldrich Cat# F1804/https://www.sigmaaldrich.cn/CN/zh/product/sigma/f1804
Mouse anti-Rab5 Santa Cruz Biotechnology Cat# sc-46692/https://www.scbt.com/p/rab-5-antibody-d-11?requestFrom=search
Mouse anti-Rab7 Santa Cruz Biotechnology Cat# sc-376362/https://www.scbt.com/p/rab-7-antibody-b-3?requestFrom=search
Mouse anti-EEA1 Santa Cruz Biotechnology Cat# sc-365652/https://www.scbt.com/p/eea1-antibody-e-8?requestFrom=search

	Mouse anti-LAMP1 Santa Cruz Biotechnology Cat# sc-20011/https://www.scbt.com/p/lamp-1-antibody-h4a3?requestFrom=search<br>Mouse anti-WWP2 Santa Cruz Biotechnology Cat# sc-398090/https://www.scbt.com/p/aip2-antibody-a-3?requestFrom=search

Eukaryotic cell lines

Policy information about cell lines and Sex and Gender in Research

Cell line source(s)	786-O, 293T, 4T1, and MDA-MB-231 cells were obtained from the Cell Bank of the Chinese Academy of Science (Shanghai, China). Renca cells were purchased from ATCC.
Authentication	All cell lines have been authenticated by short tandem repeat (STR) fingerprinting.
Mycoplasma contamination	Cell lines were routinely tested for mycoplasma and were negative.
Commonly misidentified lines<br>(See ICLAC register)	No commonly misidentified cell lines were used.

Animals and other research organisms

Policy information about studies involving animals; ARRIVE guidelines recommended for reporting animal research, and Sex and Gender in Research

Laboratory animals	For in vivo experiments with Renca cells, 6 to 8-week-old male BALB/c mice were used. For in vivo experiments with 786-O cells, 8-week-old male NOD/SCID mice were used. 6 to 8-week-old female BALB/c mice were used in the experiments with 4T1 cells. Mice were housed in barrier facilities on a 12 h light/dark cycle at temperature 18-22 degree and humidity 50-60%.
Wild animals	This study does not involve wild animals.
Reporting on sex	The findings were independent of sex. Female mice were selected for the breast cancer-related in vivo study.
Field-collected samples	This study does not involve samples collected from the field.
Ethics oversight	The study is compliant with all relevant ethical regulations regarding animal research. All animal studies were conducted according to the guidelines of the Institutional Animal Care and Use Committee of Nanjing Drum Tower Hospital.

Note that full information on the approval of the study protocol must also be provided in the manuscript.
