# Supplementary material for: Lysosomal protein transmembrane 5 promotes lung-specific metastasis by regulating BMPR1A lysosomal degradation
Source: Nat Commun. 2022 Jul 16;13:4141. doi: 10.1038/s41467-022-31783-6 (PMC9288479; doi:10.1038/s41467-022-31783-6)

Fig. 2d

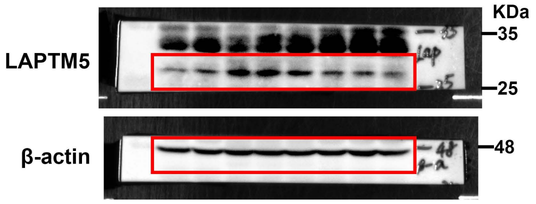

Fig. 2f

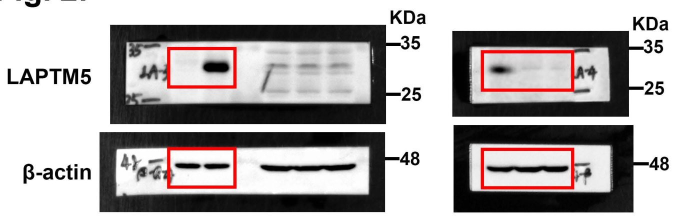

Fig. 4d

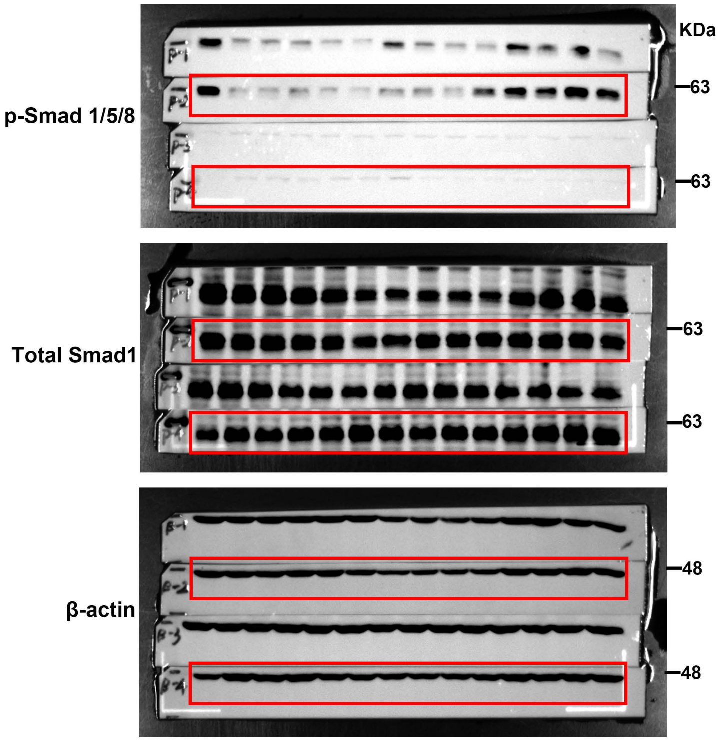

Fig. 4f

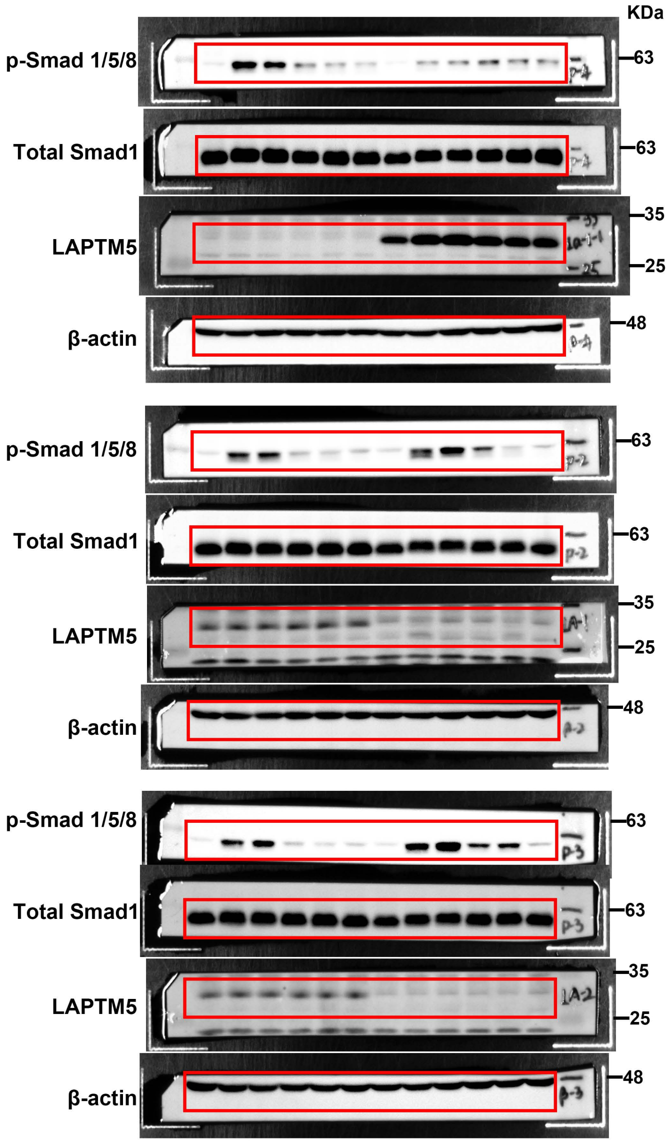

Fig. 4j + Supplementary Fig. 6g

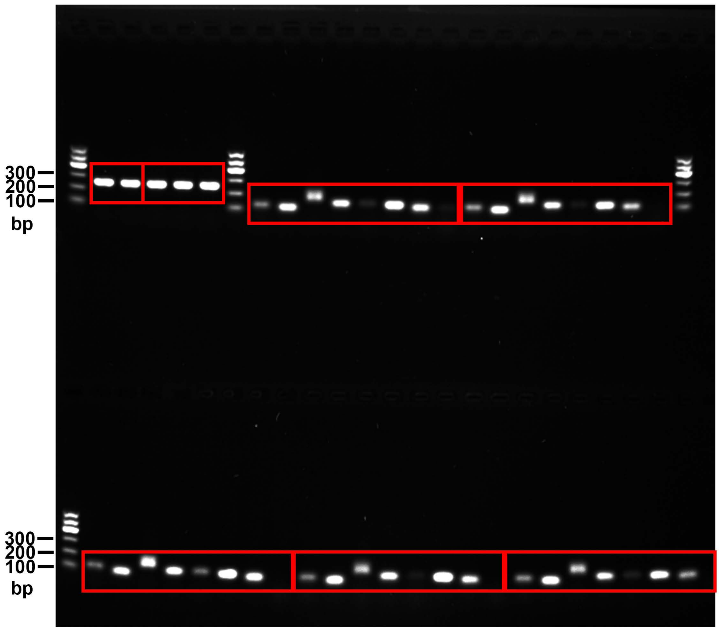

Fig. 4k

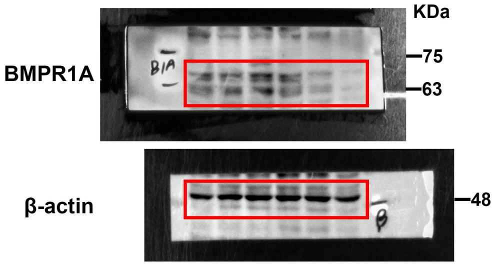

Fig. 5f

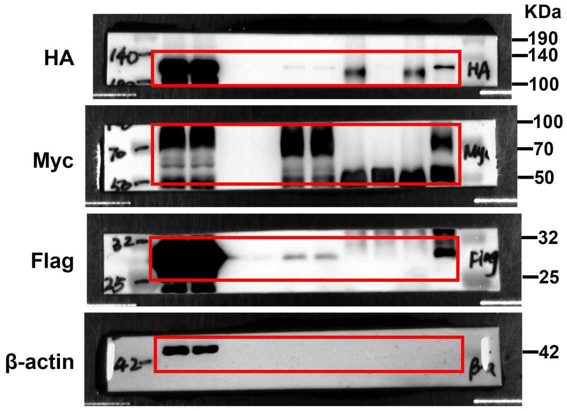

Fig. 5g

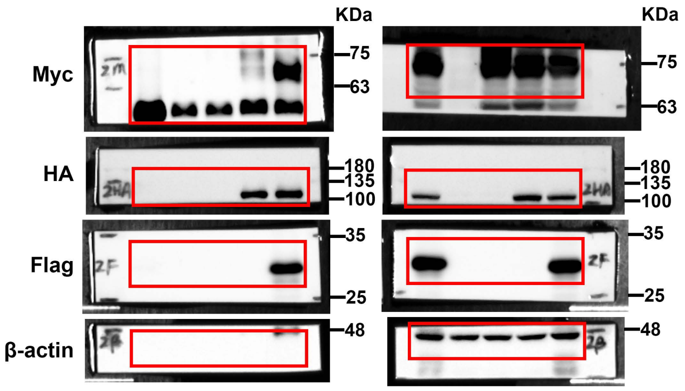

Fig. 5i

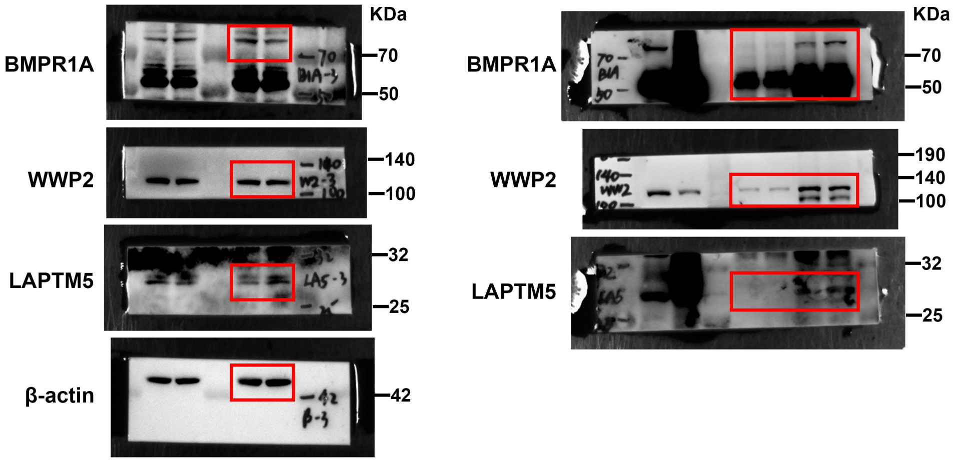

Fig. 5k

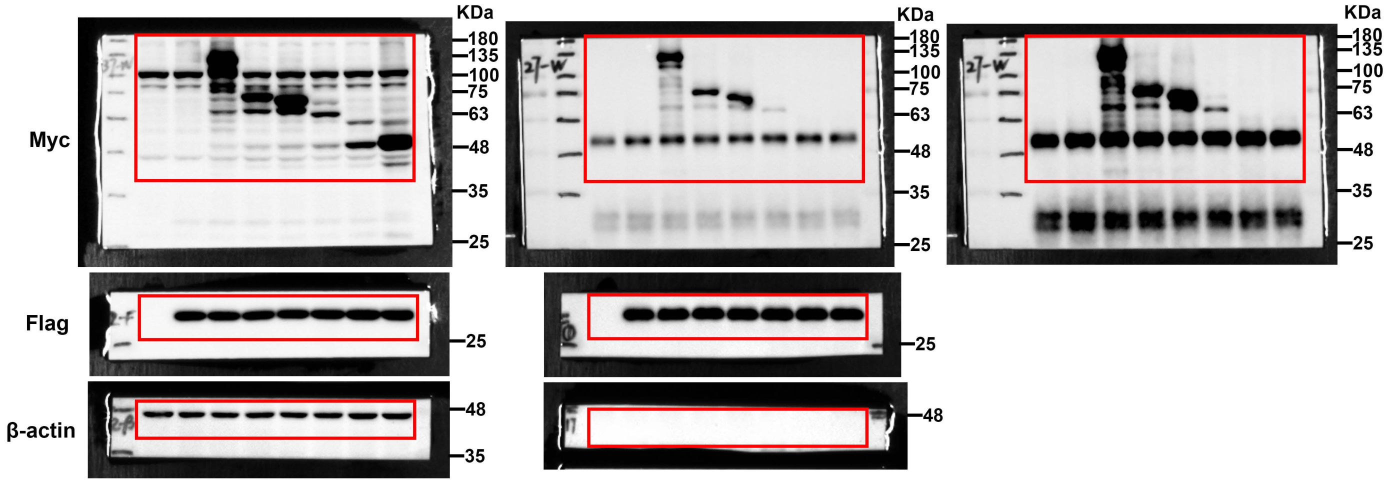

Fig. 6a

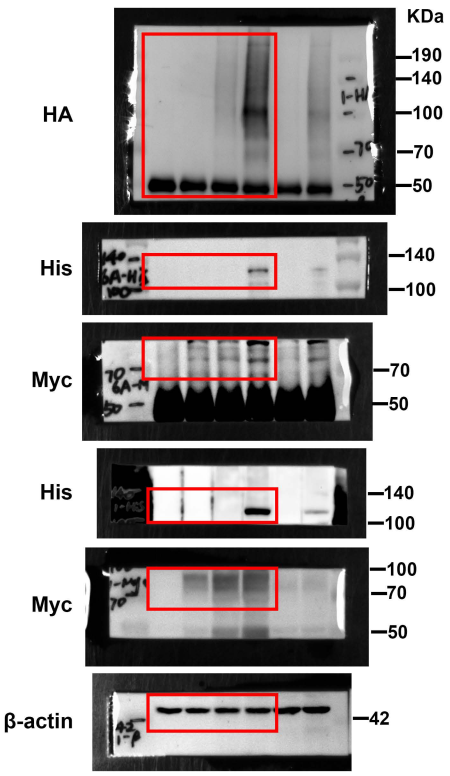

Fig. 6b

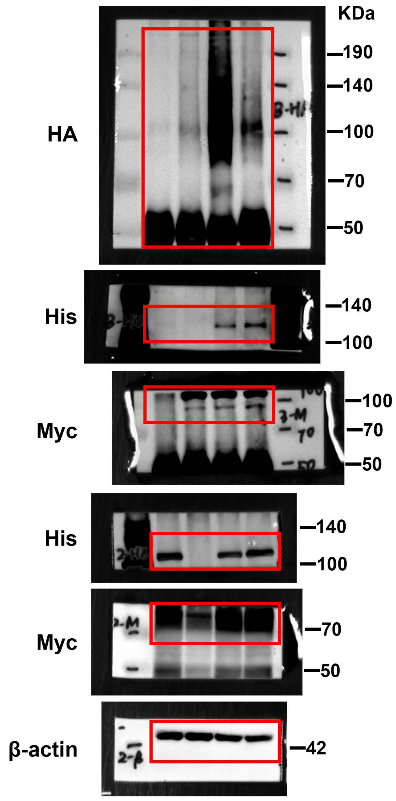

Fig. 6c

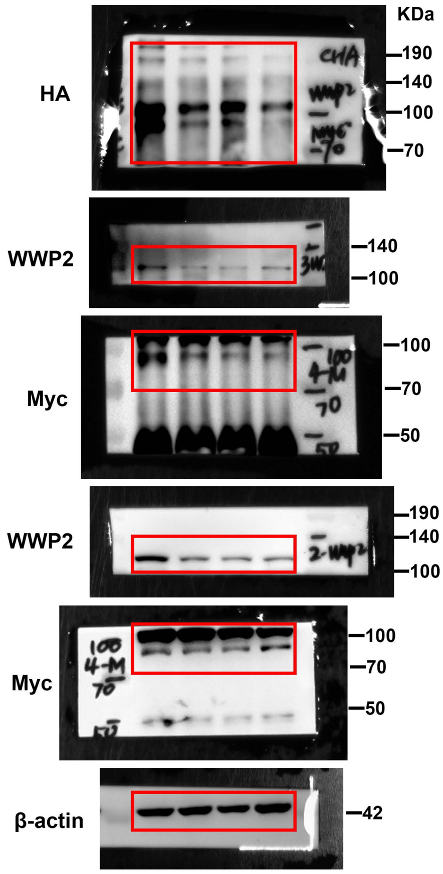

Fig. 6d

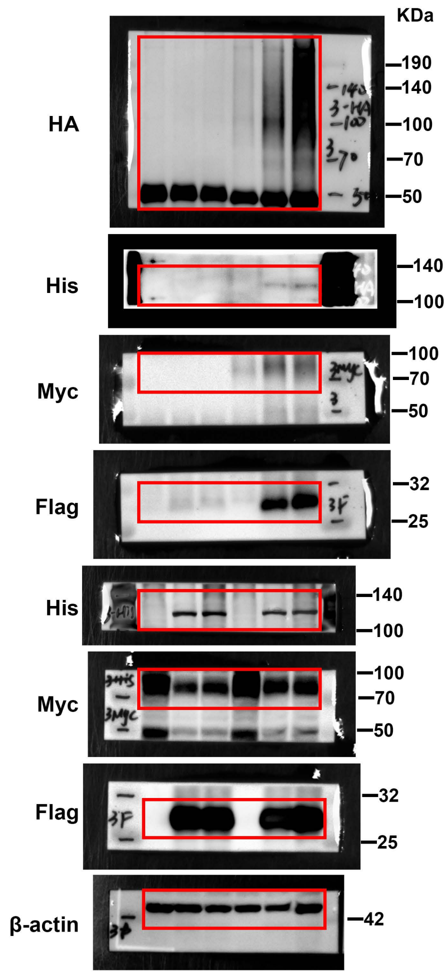

Fig. 8e

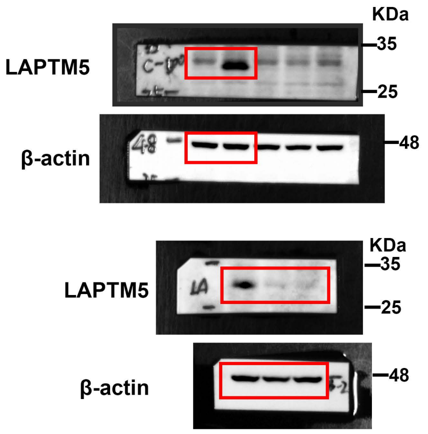

Supplementary Fig. 2d

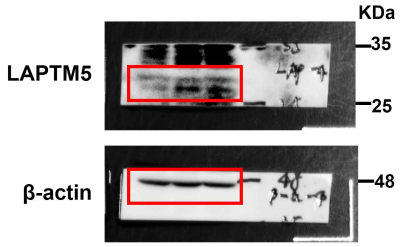

Supplementary Fig. 2e

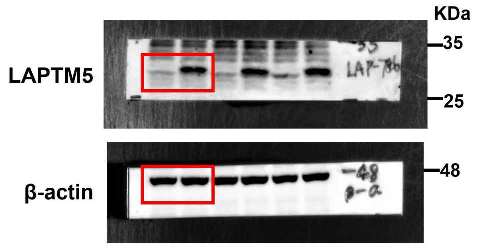

Supplementary Fig. 2g

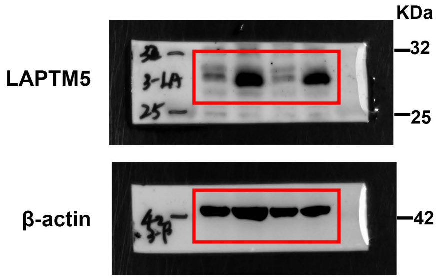

Supplementary Fig. 3d

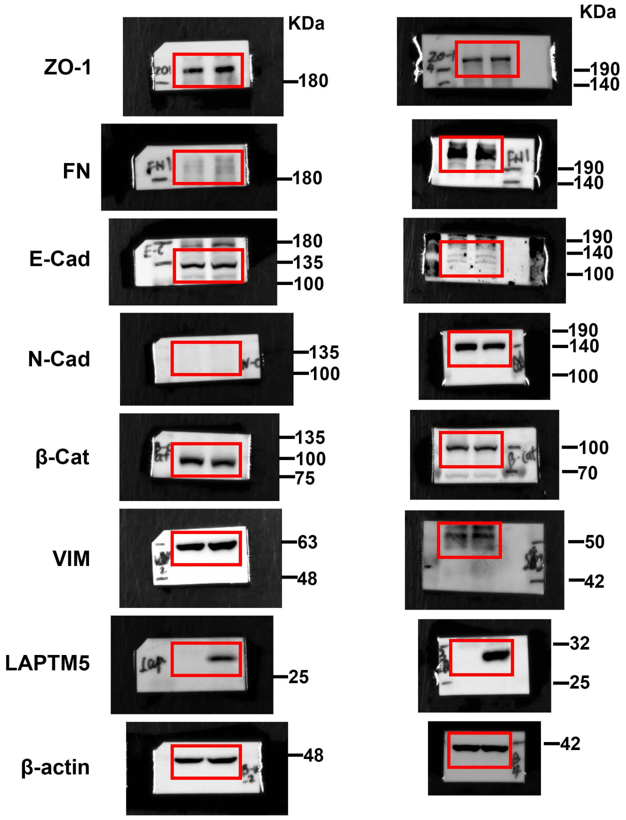

Supplementary Fig. 3k

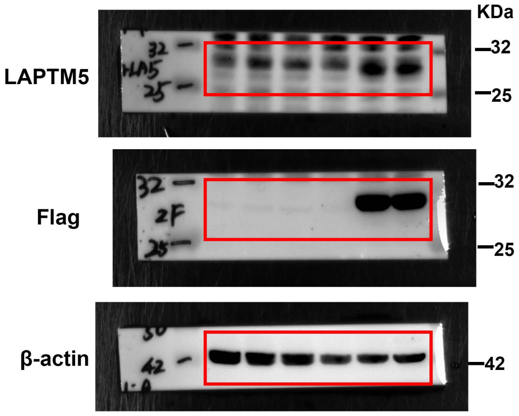

Supplementary Fig. 6b

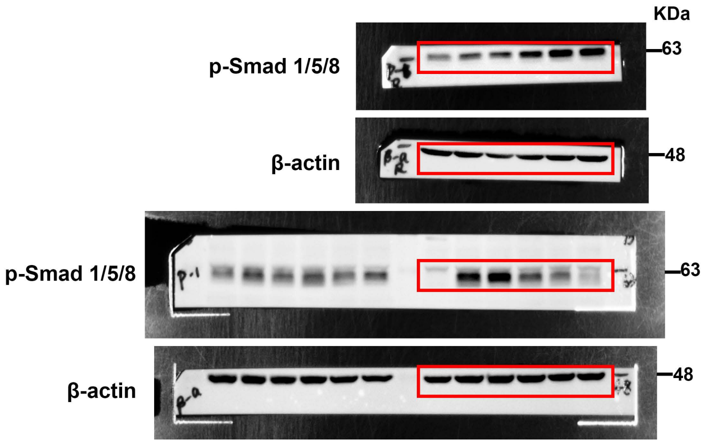

Supplementary Fig. 6c

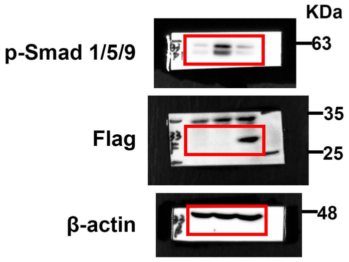

Supplementary Fig. 6d

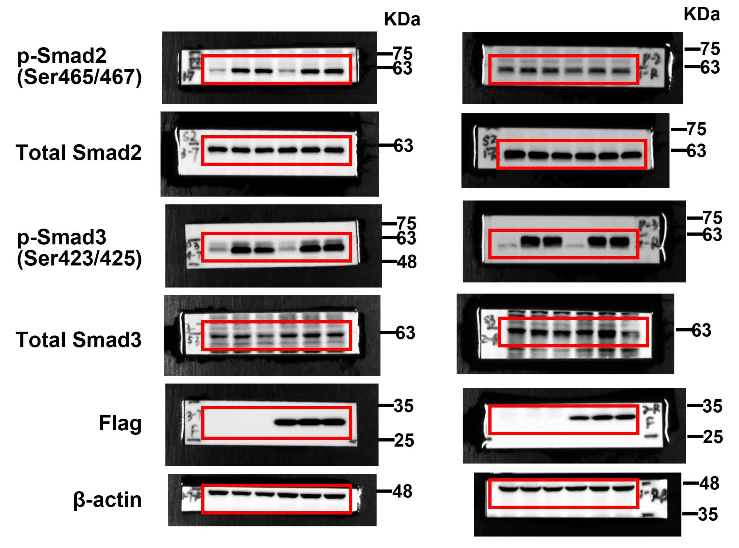

Supplementary Fig. 6e

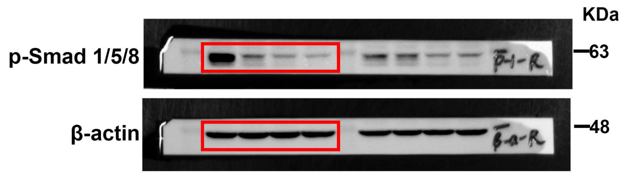

Supplementary Fig. 6g

(See Fig. 4j)

Supplementary Fig. 6h

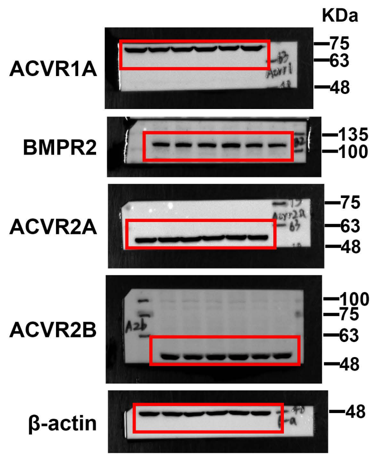

Supplementary Fig. 6i

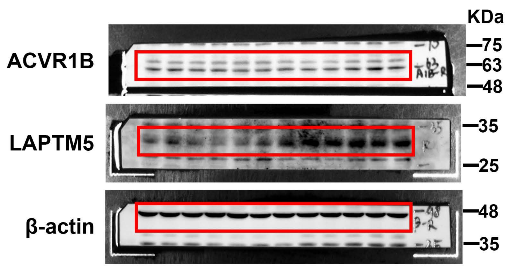

Supplementary Fig. 6j

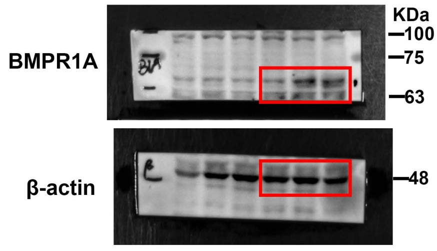

Supplementary Fig. 6l

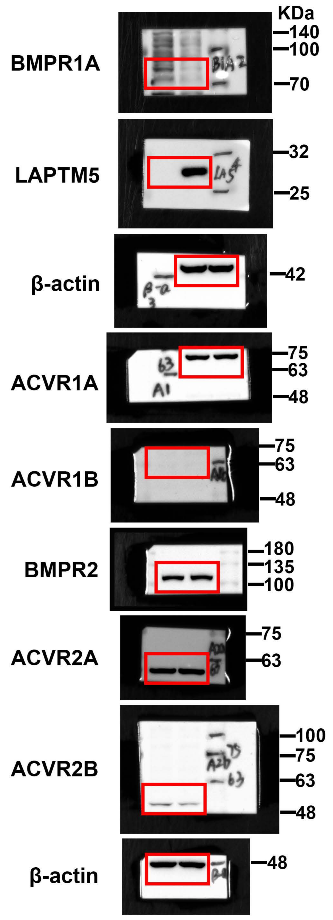

Supplementary Fig. 7b

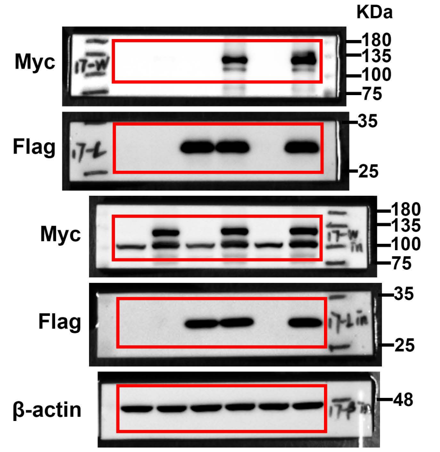

Supplementary Fig. 7c

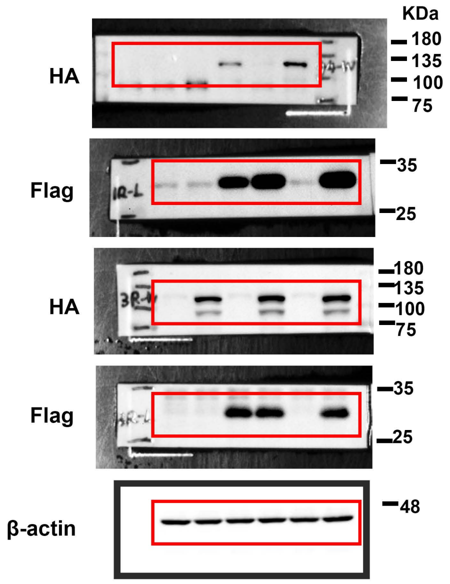

Supplementary Fig. 7d

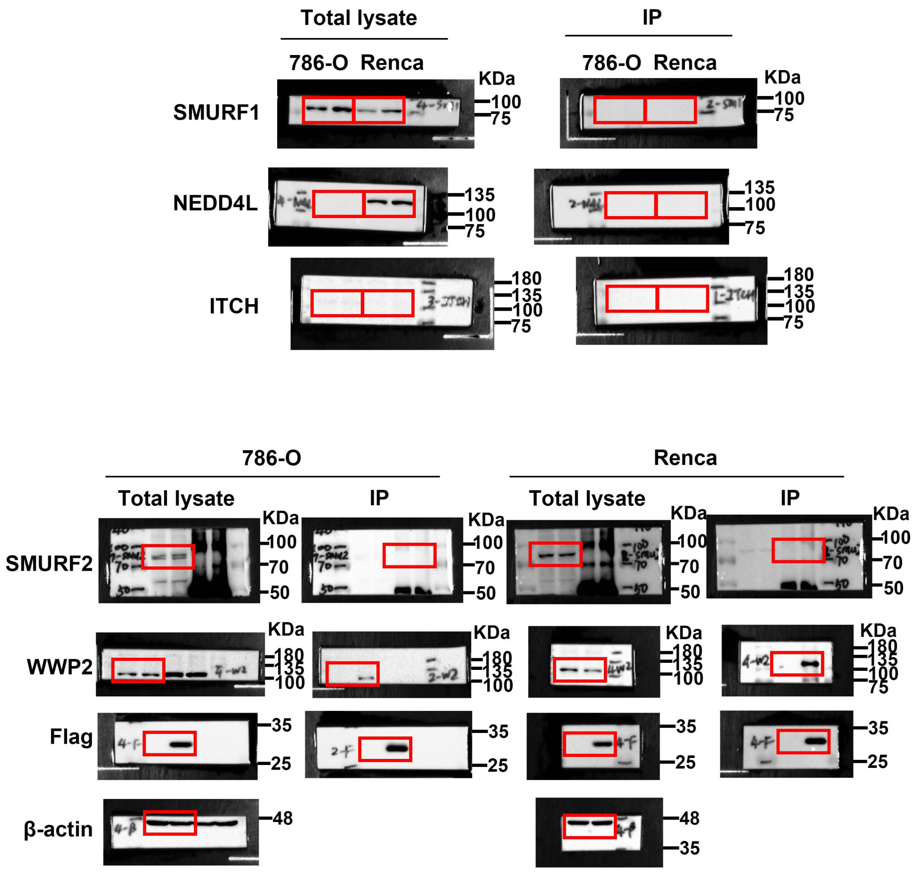

Supplementary Fig. 7f

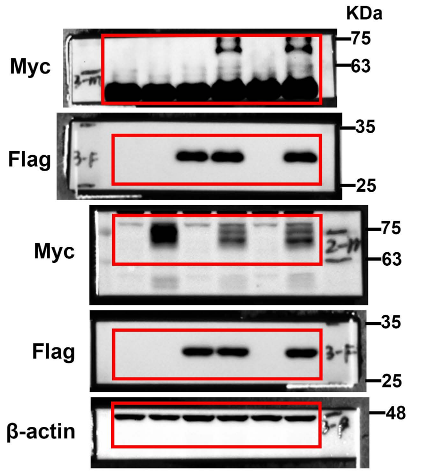

Supplementary Fig. 7g

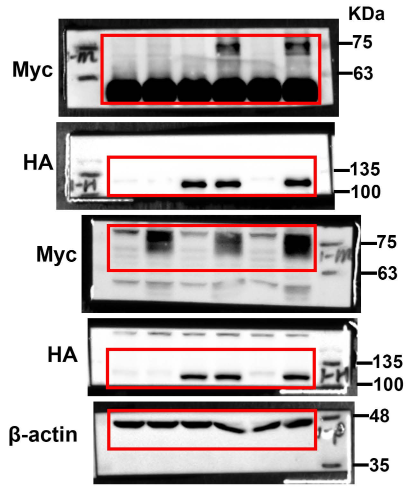

Supplementary Fig. 7i

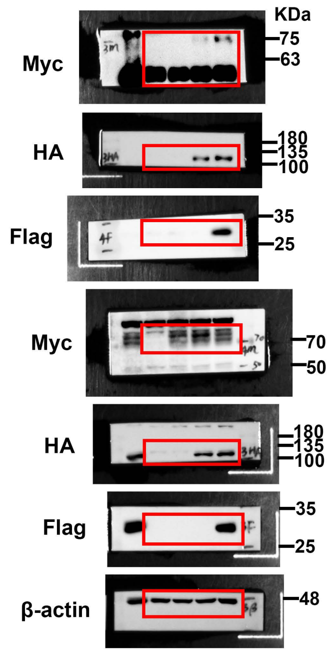

Supplementary Fig. 8a

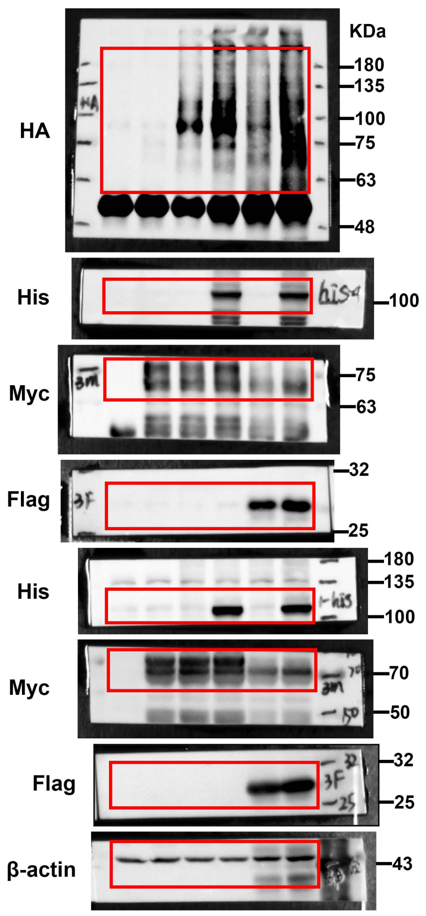

Supplementary Fig. 8b

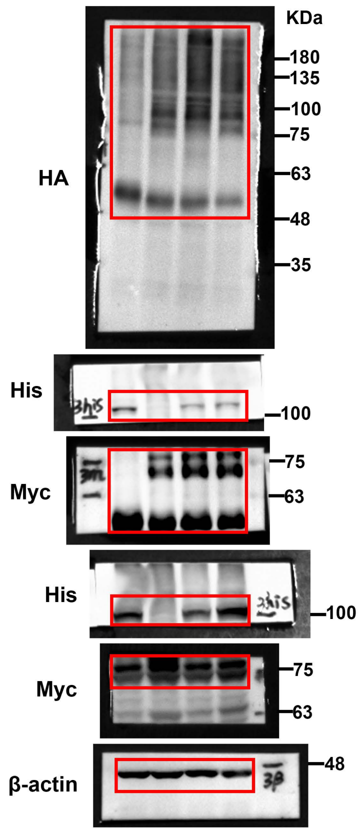

Supplementary Fig. 8c

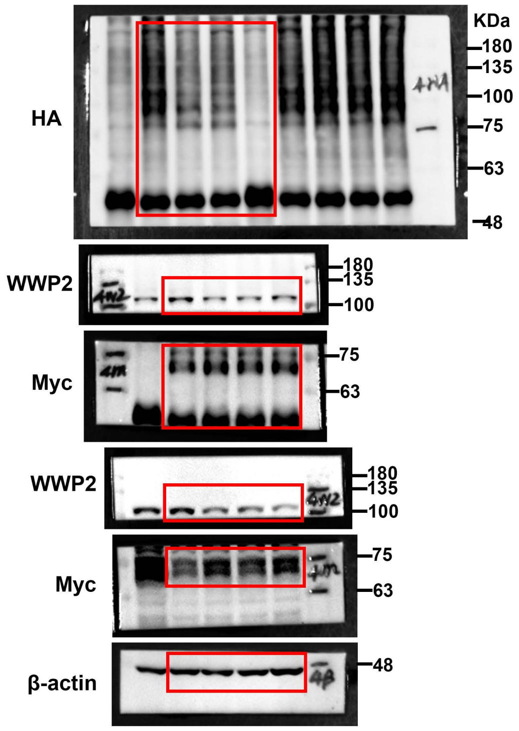

Supplementary Fig. 10c

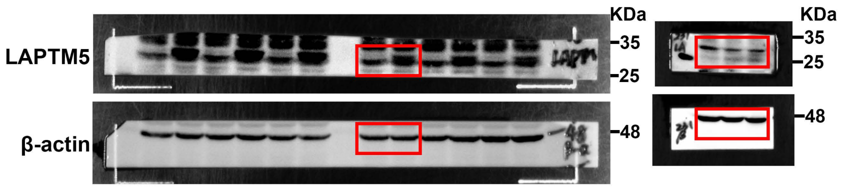

Supplementary Fig. 8d

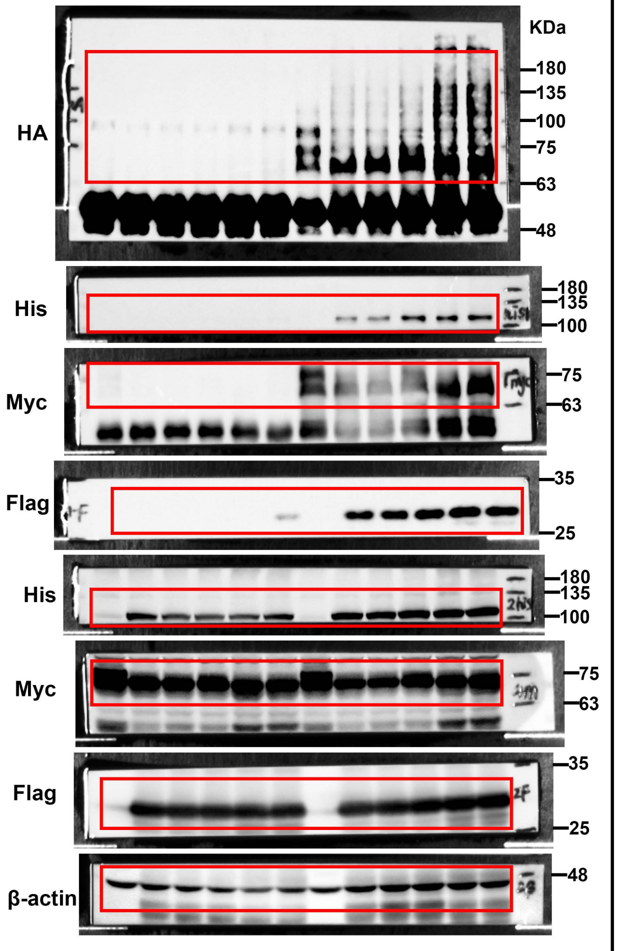

Supplementary Fig. 10g

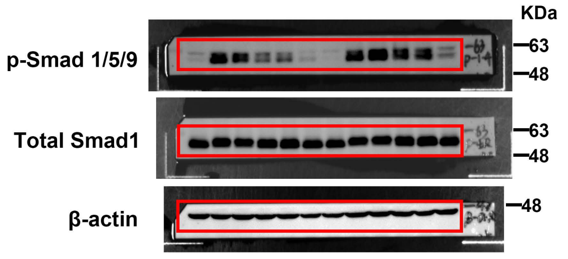

Supplementary Fig. 10h

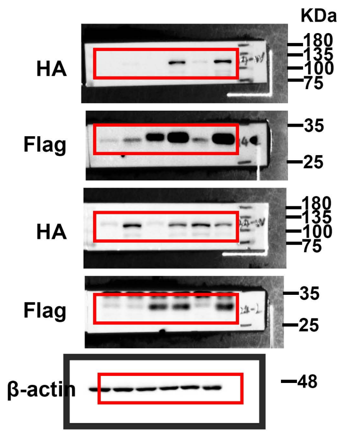

Supplementary Fig. 10l

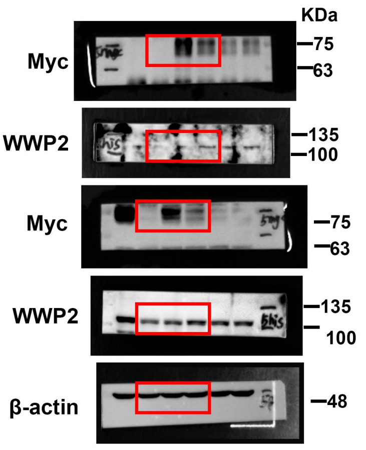

Supplement: Supplementary file 12 — Source Data [file 41467_2022_31783_MOESM12_ESM.zip › Source Data.pdf]
